# Disease avoidance threatens social cohesion in a large-scale social networking experiment

**DOI:** 10.1038/s41598-023-47556-0

**Published:** 2023-12-18

**Authors:** Hendrik Nunner, Vincent Buskens, Rense Corten, Casper Kaandorp, Mirjam Kretzschmar

**Affiliations:** 1 Institute for Multimedia and Interactive Systems (IMIS), University of Lübeck, Lübeck, Germany; 2 Department of Sociology/ICS, Utrecht University, Utrecht, The Netherlands; 3 Centre for Complex System Studies (CCSS), Utrecht University, Utrecht, The Netherlands; 4 Information and Technology Services (ITS), Utrecht University, Utrecht, The Netherlands; 5 Julius Center for Health Sciences and Primary Care, University Medical Center Utrecht, Utrecht University, Utrecht, The Netherlands

**Keywords:** Infectious diseases, Human behaviour, Epidemiology

## Abstract

People tend to limit social contacts during times of increased health risks, leading to disruption of social networks thus changing the course of epidemics. To what extent, however, do people show such avoidance reactions? To test the predictions and assumptions of an agent-based model on the feedback loop between avoidance behavior, social networks, and disease spread, we conducted a large-scale (2,879 participants) incentivized experiment. The experiment rewards maintaining social relations and structures, and penalizes acquiring infections. We find that disease avoidance dominates networking decisions, despite relatively low penalties for infections; and that participants use more sophisticated strategies than expected (e.g., avoiding susceptible others with infectious neighbors), while they forget to maintain a beneficial network structure. Consequently, we observe low infection numbers, but also deterioration of network positions. These results imply that the focus on a more obvious signal (i.e., infection) may lead to unwanted side effects (i.e., loss of social cohesion).

## Introduction

The literature contains numerous examples of individuals adapting behavior to lower their health risks (for reviews, see^1,2^). Avoidance behavior, such as avoiding large crowds or public transport during an epidemic, or avoiding others who can be a source of infections, is a typical reaction to lower the personal probability of acquiring an infectious disease^3–5^. Additionally, the extent to which a person avoids others depends on individual risk perceptions^3,6,7^. That is, the higher people perceive the likelihood to catch a disease, and the more severe these people perceive the disease to be, the more likely they are to engage in avoidance behavior. Although risk perception is an individual characteristic, the composition of risk perceptions in a social network may influence disease spread^8–10^. Furthermore, studies on epidemics in social networks have shown that the existence of clusters (densely connected areas within a network) can mitigate disease spread^11–13^.

We believe that a more profound understanding of the interdependency between avoidance behavior and disease spread in social networks is crucial for the design of effective and efficient *non-pharmaceutical interventions* (*NPIs*), such as rules for authority imposed physical distancing. For this purpose, we have developed the *Networking during Infectious Diseases Model* (*NIDM*^14^), an individual-based network model for infectious disease transmission. The NIDM defines network behavior, that is decisions to create, maintain, and break a social relation, as the trade-off between the benefits (e.g., affection, social capital, sense of belonging), costs (e.g., time, effort), and perceived risks of an infection (e.g., symptoms, hospitalization, absence from work) a social relation creates. Simulations with agents that myopically maximize the utility resulting from this trade-off produce non-linear dynamics that are hard to predict. That is, even small behavioral changes on the individual level can have large group-level effects, such as delaying an outbreak or preventing it entirely^14^. Furthermore, simulations with agents that differ in their perception of risk suggest that clusters composed of agents showing similar levels of avoidance behavior may enhance the mitigating effect of network clustering^15^.

Although agent-based models have been powerful tools for modeling health behavior and disease spread (for a review, see^16^), their insights are usually based on computer simulations making specific assumptions on individual decision-making that need empirical scrutiny. For example, humans have cognitive limits and their decisions are typically influenced by social preferences^17^. Experiments can thus reveal whether human decision makers produce the same dynamics or whether dynamics change due to the models’ simplifying assumptions^18^.

One methodological approach often adopted to probe these dynamics is the use of *incentivized experiments*. Participants of such incentivized experiments typically seek to optimize personal or joint benefits by selecting the most rewarding actions in a given situation^17^. Rewards, however, depend on the combined actions of the participants, so that decisions are made under uncertainty. To give an example, Woike et al.^19^ used a large-scale incentivized experiment to show that the effectiveness of behavioral interventions during an epidemic depends on the type of information shared with the participants (i.e., normative, informational). That is, reminding participants each round that they ought to adhere to the distancing rules to protect themselves and others was the most effective, while merely providing information on the actions of others were the least effective measures to minimize the number of infections. Importantly, the use of monetary rewards in these incentivized experiments ensures controlled variation and provides participants with a universally understood incentive, simulating real-world motivations^20^. These rewards not only facilitate precise measurement and control over the level of incentive but also guarantee consistency in the results. Furthermore, by tying real consequences to decisions through monetary stakes, participants are genuinely engaged, reflecting real-world scenarios where decisions have tangible outcomes.

### The experiment

**Figure 1:**
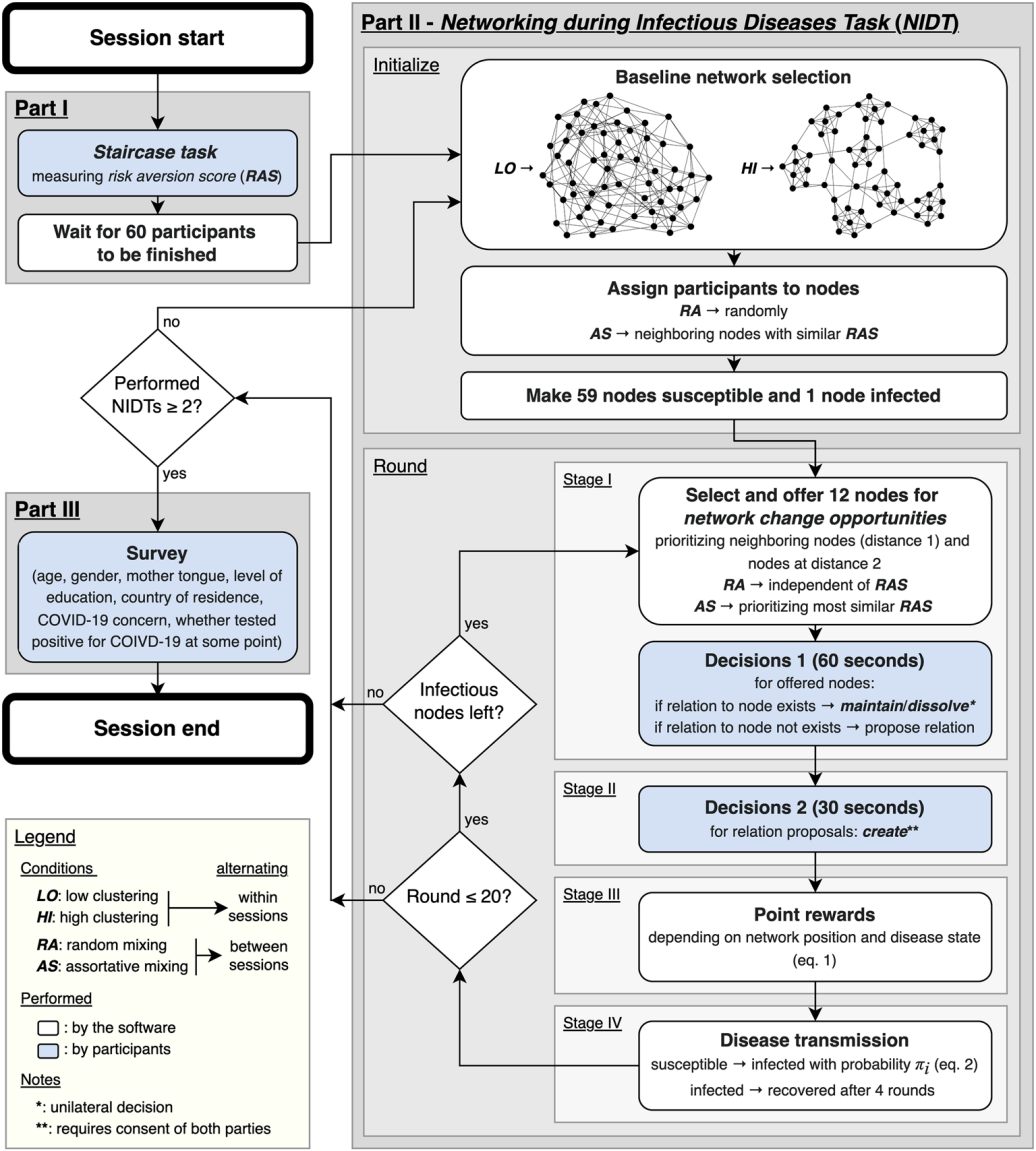
Flowchart of the experiment.

To study the feedback loop between avoidance behavior, network properties, and spread of infectious diseases, we developed a large-scale incentivized social networking experiment, consisting of three parts (see Fig. 1).

First, the *staircase task*^21,22^ consisting of five subsequent binary choices between a guaranteed reward and a 50:50 chance to win a higher reward. The more often a person opts for the guaranteed reward, although rewards for gambling increase, the more risk-averse that person is considered to be. Based on the threshold when a participant prefers gambling over a guaranteed return, we defined a *risk aversion score* per participant ranging from 0.0 to 2.0 (<1.0: risk-seeking, 1.0: risk-neutral, >1.0: risk-averse).

**Figure 2:**
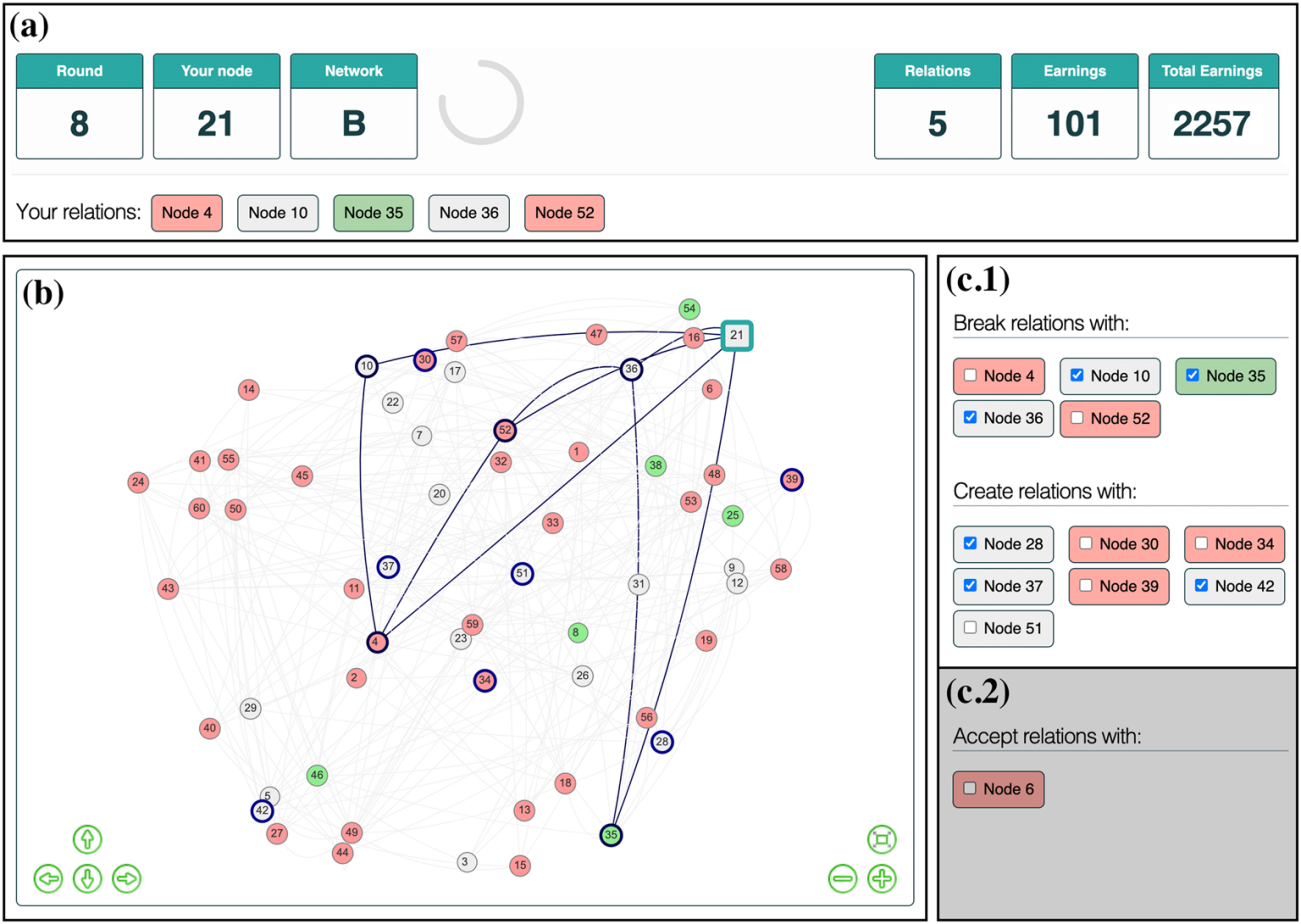
Graphical user interface (GUI) of the* Networking during Infectious Diseases Task (NIDT)*. The GUI consists of three parts: the information bar (**a**), the network viewer (**b**), and the interaction panel **(c)**. On the left, the information bar (**a**) shows the current round of the game, the node ID of the participant, the current network type (A: low clustering *LO*, B: high clustering *HI*), how much time is left in the form of a retracting circle (60 seconds for breaking/proposing relations, 30 seconds for accepting relation proposals), and the node IDs of the current relations. On the right, the information bar (**a**) displays how many relations the participant has and how many points were awarded in the previous round and in total. The network viewer (**b**) displays the entire network including nodes, disease states (gray: susceptible, red: infected, green: recovered), and social relations. The node of the participant is displayed as a square (here: node 21), while all other nodes are displayed as circles. Offered nodes are highlighted with a thick blue line. By clicking a node in the network, the relations of that node are highlighted with a blue line, while all other relations are shown in light gray. Node positions can be changed by clicking and dragging nodes. The interaction panel (**c**) is separated into two sub-panels. The upper sub-panel (**c.1**) is only visible in the first interactive stage of a round and shows the IDs of the nodes offered for decision opportunities. By unchecking a checkbox in the upper area of sub-panel (**c.1**), participants break an existing relationship, while not changing a checked checkbox corresponds to maintaining a relationship. By checking a checkbox in the lower area of sub-panel (**c.1**), participants propose to create a new relationship to another participant. The lower sub-panel (**c.2**) is only visible in the second interactive stage of a round and shows the node IDs of the corresponding participants that proposed to create a relation in the first interactive stage of the round. Checking a box in this section implies that the proposal to create a relation is accepted.

Second, the *Networking during Infectious Diseases Task* (*NIDT*), a round-based networking game (see Fig. 2). Each participant is assigned to one of sixty nodes in a fictitious network with edges representing social relations. Time is modeled as discrete time steps (*rounds* of the game). Each round is composed of four consecutive stages: two interactive stages of decision-making, computation of point rewards, and simulation of disease transmissions. Participants alternate between stages 1 and 2, while stages 3 and 4 are performed by the software with the corresponding elements being updated during website reload. Consequently, the NIDT constitutes an abstract test of a behavioral model describing the dynamics of social networks in the presence of infectious diseases. It therefore allows for insights into general avoidance behavior towards infectious diseases, while specific scenarios involving particular diseases or social contexts are excluded by design.

Third, a survey collecting additional control variables for each participant (age, gender, mother tongue, level of education, country of residence, COVID-19 concern, whether tested positive for SARS-CoV2 at some point).

In the following, we will provide a detailed description of the NIDT.

#### Available actions

In stage 1 of each round, the NIDT selects per participant 12 nodes representing other participants, and offers each participant the opportunity to *create*, *maintain*, or *dissolve* the corresponding social relations. The type of decision depends on whether a relation exists to the selected node or not. That is, in stage 1 of each round, a relation to a neighboring node can be either maintained or dissolved. The creation of a new relation with a non-neighboring node can be proposed to the corresponding participant. Nodes in the network that are closer to the participant’s node are prioritized by the NIDT. That is, selected nodes are on average 50% neighbors, 30% neighbors of neighbors, and 20% others.

In stage 2 of each round, the participants that received proposals to create new relations can decide whether to accept the proposals or not. Thus, dissolution of relations is a unilateral decision, while the creation of a relation requires the consent of both participants involved.

#### Point rewards

In stage 3 of each round, point rewards are awarded. Computation of points is based on theoretical considerations regarding the effects of social relations on well-being. That is, while social relations are beneficial for social well-being (e.g., affection, sense of belonging)^23^, they also produce costs in maintenance (e.g., effort, time)^24^. Additionally, infectious social relations constitute a risk to well-being, as they have the potential to cause physical harm^23^. Consequently, social networking decisions in the context of infectious diseases are based on a trade-off between the social benefits, social costs, and perceived potential health costs a social relation creates (for more details on the theoretical background of our model, see^14^).

Furthermore, costs and benefits for social relations depend on the social context. That is, in a work environment, people may prefer relations between not connected others (A is connected to B and C, while B and C are not connected to each other) to be able to control information flow^25^. In private settings, such as family and friends, people may prefer *triadic closure* (A is connected to B and C, while B and C are also connected to each other)^26^ for better social support or more leverage to enforce social norms^27^. These individual-level preferences exert scaling effects on the structure of the entire social network, by either giving rise to a large sparsely connected component or several densely connected clusters.

Based on the aforementioned considerations, the NIDT defines point rewards (or *utility*, *U*) of an individual *i* as the composition of three terms:

1 $$\begin{aligned} U_{i} = \bigg [b_{1} \cdot t_{i} + b_{2} \cdot \left( 1 - 2 \cdot \frac{\left| x_{i} - \alpha \right| }{\max \left( \alpha , 1 - \alpha \right) } \right) \bigg ] - \bigg [c_{1} \cdot t_{i} + c_{2} \cdot t_{i}^{2}\bigg ] - \bigg [\sigma \bigg ]\text {.} \end{aligned}$$

First, there are rewards ($b_{1}$) for the number of social relations ($t_{i}$) and the weighted ($b_2$) proportion of closed triads ($x_{i}$) *i* belongs to, while $\alpha$ is the preferred proportion of closed triads. This operationalization of $\alpha$ allows that the proportion of closed triads can be precisely controlled. That is, a higher $\alpha$ (e.g., close to 1.0) leads to a higher utility, if many social relations of an individual are also related to each other. Consequently, networks with strong clique formation emerge. Second, there are marginally increasing costs ($c_{1}$, $c_{2}$) for the number of social relations ($t_{i}$). That is, each additional relation creates higher costs than the previous one, so that the optimal number of social relations can be controlled. Third, there are costs for being infected ($\sigma$).

Since such a complex reward system is not only difficult to understand but also too artificial to be used in an experiment, we provided the participants with easy to understand instructions of the rules devoid of the underlying utility function (see Section 1.3 in the Supplementary Information for the complete instructions). Furthermore, we chose parameter settings resulting in easy to understand point rewards presented in a table showing exact point rewards per number of social relations and explanations of additional points for the optimal proportion of closed triads. All rules were also accessible via a pop-up window throughout the entire experiment. Participants were thus neither shown nor required to understand Eq. (1).

Parameter settings for Eq. (1) were determined using computer simulations, to ensure a combination of accessibility and interesting dynamics between social networks and disease spread (for technical details, see Section “Hypotheses and conditions”). Regarding the number of relations, we implement parameters for six relations to be most beneficial. That is, having six relations awards 100 points per round. Having more or fewer relations results in progressively lower point rewards (see Table 1). Negative rewards for number of relations were set to 0, so that having more than 11 relations did not result in a loss of points. To incentivize the maintenance of clustering of the initial networks (see Fig. 1), the NIDT awards up to 20 points depending on the setting for clustering (for details on parameter settings and conditions, see below). That is, in the low clustering setting (*LO*; $\alpha = 0.0$) 20 points are rewarded for not being part of a closed triad. In the high clustering setting (*HI*; $\alpha = 0.67$) 20 points are rewarded if two thirds of the neighbors form closed triads. The further away participants are from the optimal clustering, the fewer points they receive. Node positions in the initial networks are optimal in terms of network benefits. That is, in the absence of infection, benefits cannot be increased by adding or removing social relations. The experiment is thus focused on changing network structure under the threat of infection. Finally, 14 points are deducted for every round a participant is infected. This relatively low penalty was chosen so that it would not necessarily be beneficial to immediately dissolve a relation with a single infected alter.

**Table 1:**
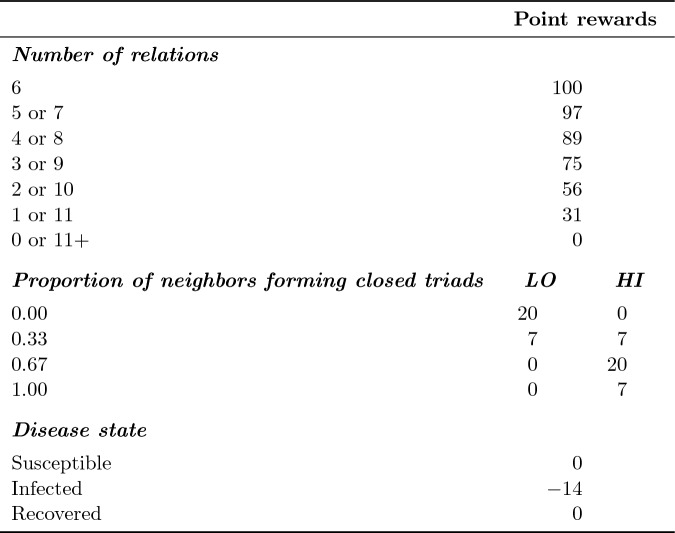
Properties and corresponding point rewards per round.

**Notes:** Point rewards are added up and rewarded each round of the game. Parameter settings (see Eq. (1)) are: First, the number of relations: $b_{1} = 1.0$, $c_{2} = 0.067$. Second, the proportions of neighbors forming closed triads. The proportions shown serve to illustrate the difference between the low (*LO*; $b_{2} = 0.5$, $\alpha = 0.0$.) and high (*HI*; $b_{2} = 0.5$, $\alpha = 0.67$) clustering settings. Third, disease state: $\sigma = 0.34$. Finally, a scaling factor of 41.55 was used to ensure easy to understand point rewards.

#### Disease transmission

In stage 4 of each round, disease transmissions from infectious to susceptible nodes via social relations are computed. The probability for a susceptible node to get infected depends on the number of infected (and thus infectious) neighbors:

2 $$\begin{aligned} \pi _{i} = 1 - (1 - \gamma )^{t_{i_{I}}}\text {,} \end{aligned}$$

with $\gamma$ denoting the probability to get infected per neighbor and $t_{i_{I}}$ the number of infected neighbors of participant *i*. We use $\gamma = 0.15$ because simulations have shown that this creates interesting dynamics, where diseases spread without infecting the entire network and agents have sufficient time to adjust their network relations to the threat of infection. Once infected, nodes recover after 4 rounds and cannot get infected again. Participants were not required to understand Eq. (2), as we provided them with a table showing the probabilities of infection per number of infected neighbors (see Section 1.3 in the Supplementary Information).

The NIDT concludes either after 20 rounds, or when no more infectious nodes are left in the network.

### Hypotheses and conditions

Hypotheses and conditions were derived based on a simulation study^15^. Furthermore, parameter settings for the conditions were determined using additional simulations (for details see Section 2.1 of the Supplementary Information). In all simulations, we used artificial agents that myopically optimized rewards provided by Eq. (1). To realize individual risk perceptions, we replaced the costs of being infected ($\sigma$) with perceived costs of being infected ($\sigma ^{r_{i}}$) depending on perceived risks of getting infected ($\pi _{i}^{2-r_{i}}$):

3 $$\begin{aligned} \bigg [\sigma \bigg ] \rightarrow \sigma ^{r_{i}} \cdot \pi _{i}^{2-r_{i}} \text {.} \end{aligned}$$

That is, the risk perception parameter $r_{i}$ ($0.0< r_{i} < 2.0$) transforms severity of the disease ($\sigma$) and probability of getting infected ($\pi _{i}$) into subjective versions of the same. Consequently, higher values for $r_{i}$ lead to higher perceived costs of being infected and higher perceived risks of contagion, and therefore correspond to higher risk aversion. A value of $r_{i} = 1.0$ corresponds to risk neutrality.

Simulations showed that susceptible agents avoid infectious agents, higher risk aversion leads to stronger avoidance of infectious agents, and stronger avoidance of infectious agents leads to smaller epidemics^15^. We therefore hypothesize:

**H1:** Infectious alters are avoided by susceptible egos.
**H2:** Higher risk aversion causes stronger avoidance of infectious alters.
**H3:** Higher risk aversion lowers the individual probability of getting infected.

To test Hypotheses H1–H3, we used risk aversion scores of participants collected from the staircase task (see Section “The experiment” for details).

In line with earlier studies (e.g.,^11–13^), our simulations suggested that epidemics are smaller in networks with multiple densely connected clusters than in networks with a more open structure. That is because relations that bridge two clusters pose a bottleneck that impedes the further spread of a disease. In addition, our simulations suggested that assortative mixing in terms of health risk perceptions, and thus the local aggregation of individuals who respond similarly to health risks, may further reduce epidemic size. Finally, our simulations suggested that the combination of assortative mixing in networks with multiple clusters produces the smallest epidemics^15^. That is because a cluster composed of predominantly risk-averse agents is likely to quickly dissolve bridges to infectious alters, protecting the entire cluster from getting infiltrated by the disease. We therefore hypothesize:

**H4:** Epidemics are smaller in networks with a higher degree of clustering.
**H5:** Epidemics are smaller in networks with a higher degree of assortative mixing regarding risk aversion.

To test Hypotheses H4 and H5, we defined four conditions (*LO:RA*, *LO:AS*, *HI:RA*, *HI:AS*) varying network clustering and assortative mixing in a two-by-two design. Network clustering is either low (*LO*) or high (*HI*) and social mixing is either random (*RA*) or assortative (*AS*) regarding risk aversion. Clustering settings were realized with two different baseline networks. That is, one network was composed of a single large cluster (*LO*), while the other contained several densely connected clusters (*HI*; see Fig. 1). To eliminate unwanted structural side effects, both networks were similar regarding average degree (both 5.93) and closeness (*LO*: 0.975, *HI*: 0.955).

While settings for clustering were communicated to the participants, settings for social mixing were not. To realize differences in social mixing, the NIDT first initialized networks with either low or high degrees of assortative mixing. That is, risk aversion scores of participants were first arranged in ascending order. Thereafter, the corresponding participants were assigned to fixed orders of nodes to attain either networks with neighboring nodes possessing mostly different risk aversion scores (*RA*) or networks with neighboring nodes possessing mostly similar risk aversion scores (*AS*). Furthermore, to maintain the level of assortative mixing, nodes for decision opportunities (creation and dissolution) were also selected based on risk aversion score. That is, to inhibit assortative mixing (*RA*), the NIDT selected nodes independent of risk aversion score (ω = 0.0, with ω denoting the probability of selecting the node most similar regarding risk aversion score). To foster assortative mixing (*AS*), the NIDT prioritized nodes most similar regarding risk aversion score (ω = 0.8).

Finally, we predefined the index cases (the initially infected node) for each condition so that the epidemic started with the node that is closest to average degree, average local clustering coefficient, and average risk aversion score. We used neutral, not emotionalizing language to explain the rules of the NIDT (see Section 1.3 of the Supplementary Information). That is because we intend to study risk perceptions as an analytical heuristic in decision-making. Furthermore, although the experiment was conducted during the COVID-19 pandemic, we did not use COVID-19 as context or motivation. We believe that this facilitates the repeatability of the experiment and the comparability of the results for other conditions or parameter settings.

## Results

Our results are based on data collected from 48 experimental sessions, with each session composed of one staircase task, two NIDTs (clustering alternating within each session, social mixing alternating between sessions), and a final survey (age, gender, mother tongue, level of education, country of residence, COVID-19 concern, whether tested positive for SARS-CoV2 at some point). Consequently, we analyzed a total of 96 NIDTs, with 24 NIDTs per experimental condition, and a total number of 711,159 networking decisions made by 2,879 participants.

### The role of disease avoidance in decision-making

Table 2 shows the proportion of decisions made (accepted decision opportunities) to decision opportunities (nodes offered by the NIDT in stage 1 and relation proposals by other participants in stage 2 of a round). Furthermore, Table 2 shows the proportion of decisions made that were rewarding in terms of increasing or maintaining point rewards to decisions made that lowered point rewards (in parentheses). The data is divided by decision type (*Create*, *Not create*, *Dissolve*, *Maintain*) and disease states (**S**usceptible, **I**nfected, **R**ecovered) of the ego (decision maker) and alter (subject of the decision).

**Table 2:**
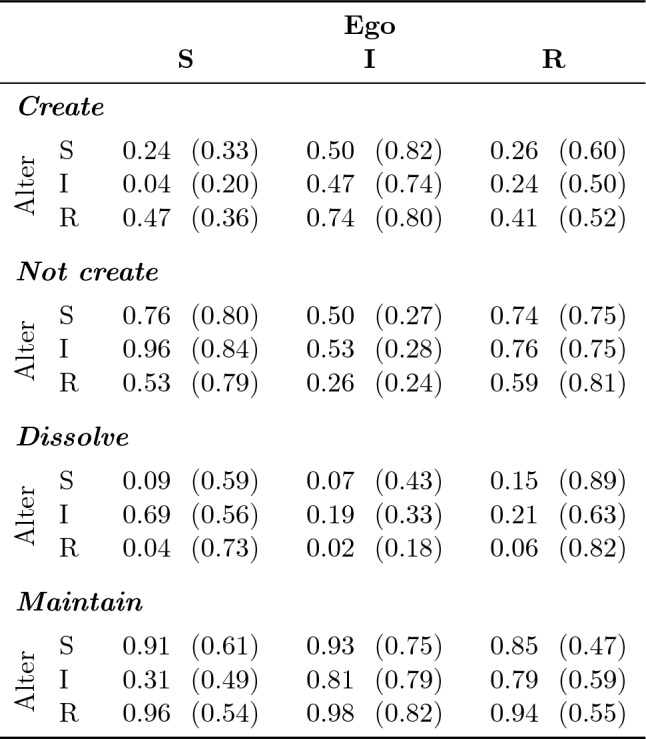
Proportions of specific decisions made given specific decision opportunities, and the proportion of those decisions with rewards higher than the rewards of the opposite decision.

Numbers without parentheses give the proportion of decisions that were made for all decision opportunities in that cell; numbers within parentheses give the proportion of the decisions that were made that led to higher rewards than the opposite decision. For example, when susceptible egos had the opportunity to create a relation to susceptible alters, they were willing to make that decision in 24% of all opportunities. In 33% of these decisions to create a relation, the rewarded points were higher than if they had not decided to create that relation. Furthermore, *Create* and *Not create* as well as *Dissolve* and *Maintain* are opposite decisions for the same opportunities: *Create* $\rightarrow$  the combination of accepted opportunities to propose a relation in stage 1 and accepted relation proposals in stage 2 of a round; *Not create* $\rightarrow$  the combination of declined opportunities to propose a relation in stage 1 and accepted relation proposals in stage 2 of a round; *Dissolve* $\rightarrow$  accepted opportunity to dissolve a relation in stage 1 of a round; *Maintain* $\rightarrow$  declined opportunity to dissolve a relation in stage 1 of a round.

The data reveal that infected alters are avoided the most. That is, relations to infected alters were created the least, while existing relations to infected alters were dissolved the most. Although this effect can be found independent of disease states of the ego, it is strongest for egos that are susceptible and thus at risk of getting infected themselves. That is, susceptible egos dissolved 69% of the relations if the offered node was infected, compared to 9% and 4% if the offered node was susceptible or recovered. The decision of susceptible egos to avoid infectious alters, however, was rewarding for only about half of the decisions (56%). The average decision of infected egos to create and maintain relations, in contrast, was among the most rewarding. This is likely an effect of the lowered average degree of infected nodes, which dropped from an average of more than seven relations in the round before acquiring an infection (*M* $=7.4$, *SD* $=3.1$) to less than 3 relations for the entire period of being infected (*M* $=2.8$, *SD* $=1.99$; see also Figure S5 in the Supplementary Information). Creating and maintaining relations therefore allows compensating for points lost as a result of social isolation. Thus, these data suggest that while susceptible egos make decisions in order to avoid the disease at a potential cost of point rewards, infected egos make decisions in order to minimize losses due to being socially isolated.

**Figure 3:**
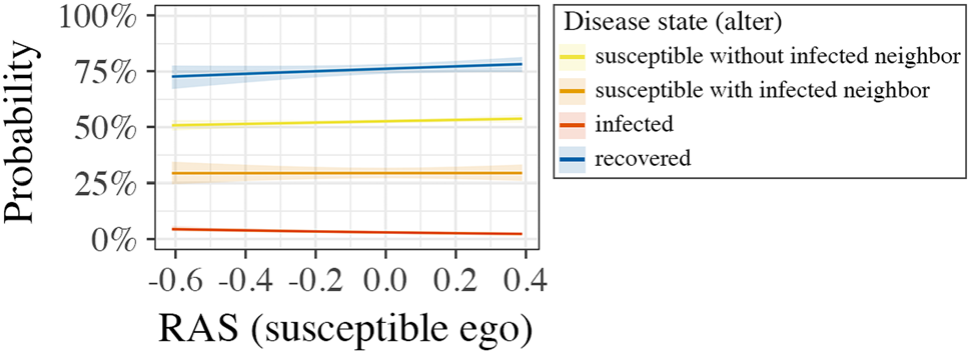
Marginal effects of risk aversion score of susceptible egos and disease states of alters on the probability of a relation being desirable. Risk aversion score is mean centered at *M* =1.22 (*SD* =0.46), denoting that the average participant was slightly risk-averse (<1.0: risk-seeking, 1.0: risk-neutral, >1.0: risk-averse). Risk aversion score refers to the ego, the node, to describe the degree of desire for a relation. Colors denote the disease state of the alter, the subject of the desire for a relation.

As Table 2 suggests that disease states influence how much one relation is preferred over another, we performed logistic regressions on the factors contributing to the attractiveness of a relation (Table 3). A relation being attractive is represented by a binary variable composed of decisions to create a relation (proposals in stage 1 and accepted proposals in stage 2 of a round) and declined opportunities to dissolve a relation. Based on the regression analysis, Fig. 3 reveals that susceptible egos favor relations to recovered alters, followed by susceptible alters without infected neighbors, then susceptible alters with infected neighbors, and finally infected alters. The interaction effect between susceptible egos, infected alters, and risk aversion score (also visible by the negative slope of the red line in Fig. 3), reveals that the more risk-averse the ego is, the less attractive an infected alter gets. Furthermore, Table 3 shows that recovered alters are the most attractive, while infected alters are among the least attractive relations for all egos.

**Table 3:** Factors contributing to the attractiveness of a relation.

Constant	5.866*** (0.074)	
**Disease states**		
Ego^S^ - Alter^S^ without infected neighbor	−0.103* (0.059)	
Ego^S^ - Alter^S^ with infected neighbor	−1.070*** (0.061)	
Ego^S^ - Alter^I^	−3.728*** (0.067)	
Ego^S^ - Alter^R^	1.070*** (0.063)	
Ego^I^ - Alter^S^ without infected neighbor	0.200*** (0.062)	
Ego^I^ - Alter^S^ with infected neighbor	−0.014 (0.079)	
Ego^I^ - Alter^I^	−1.347*** (0.094)	
Ego^I^ - Alter^R^	1.949*** (0.114)	
Ego^R^ - Alter^S^ without infected neighbor	−0.886*** (0.057)	
Ego^R^ - Alter^S^ with infected neighbor	−0.813*** (0.083)	
Ego^R^ - Alter^I^	−1.560*** (0.084)	
Ego^R^ - Alter^R^ (reference)		
**Risk aversion**		
Risk aversion score (RAS)	0.185 (0.172)	
Ego^S^ - Alter^S^ without infected neighbor $\times$ RAS	-0.037 (0.161)	
Ego^S^ - Alter^S^ with infected neighbor $\times$ RAS	−0.130 (0.179)	
Ego^S^ - Alter^I^ $\times$ RAS	−0.833*** (0.235)	
Ego^S^ - Alter^R^ $\times$ RAS	0.182 (0.199)	
**Network properties**		
Degree	−0.492*** (0.007)	
Degree^2^	0.020*** (0.001)	
Degree of alter	−0.043*** (0.001)	
Clustering (*HI*)	0.026 (0.032)	
*HI* $\times$ ECC^†^	−10.258*** (0.106)	
*LO* $\times$ ECC^†^	6.655*** (0.137)	
**Decision opportunity type**		
Creation opportunity in stage 1 of a round	−4.126*** (0.010)	
Creation opportunity in stage 2 of a round	−3.299*** (0.015)	
Number of offers by opportunity type	−0.098*** (0.003)	
Log Likelihood	−286,474	
AIC	573,006	
BIC	573,339	
Observations	711,159	
Number of groups (rounds)	52,650	
Number of groups (nodes)	5758	
Number of groups (games)	96	
Variance (rounds)	0.28	
Variance (nodes)	0.87	
Variance (games)	0.01	

The numbers describe a four-level random intercept logistic regression model (level 4: games, level 3: nodes, level 2: rounds, level 1: decisions) of whether a relation is attractive (0: declined opportunities to create a relation in stage 1 and 2 of a round, and accepted opportunities to break a relation, 1: accepted opportunities to create a relation in stage 1 and 2 of a round, and declined opportunities to break a relation). Refer to Table S10 in the Supplementary Information for a comparison of models by different decision opportunities.

***p < 0.01, **p < 0.05, *p < 0.1, SEs in parentheses.

^†^*ECC*: Expected Change in Clustering.

In summary, we find support for Hypotheses H1 and H2. That is, infected alters are the least desirable nodes for susceptible egos and higher risk aversion causes stronger avoidance of infectious alters. Our results, however, go beyond what could be expected from our model. That is, on the one hand, avoidance behavior towards infected alters can also be observed for infected and recovered egos. On the other hand, susceptible nodes that have infectious neighbors are avoided by susceptible egos. As a result, infected nodes are getting more strongly isolated in the experiment than in our simulations. That is, infected nodes in the experiment have on average 2.8 relations, while infected nodes in the simulations have on average 5.6 relations (see also Figure S5 in the Supplementary Information). This stronger isolation of infected nodes in the experiment has direct implications for disease spread on the group-level.

### Group- and individual-level effects

**Figure 4:**
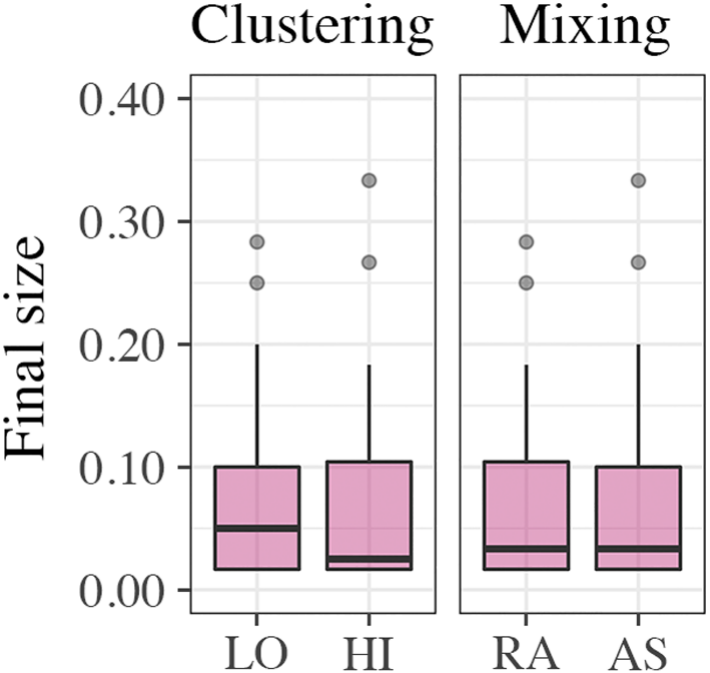
Final epidemic size by clustering and social mixing. Box-and-whisker plots show the median, interquartile range, minimum, maximum, and outliers of final size by settings for clustering and social mixing. Final size describes the proportion of nodes that got infected at some point during a single installment of the NIDT.

Figure 4 shows that, on average, final epidemic size (the proportion of infected nodes during a single installment of the NIDT) did not differ between settings for clustering (*LO*: *Mdn* =0.05, *HI*: *Mdn* =0.03) and mixing (*RA*: *Mdn* = 0.04, *AS*: *Mdn* = 0.04). Wilcoxon rank-sum tests confirmed that the differences were not statistically significant for clustering (*Z* = −1.31, *p* = 0.19) and mixing (*Z* = −0.06, *p* = 0.95). Because these results do not show any discernible differences between the different settings, we can neither confirm nor reject our group-level hypotheses regarding clustering and assortative mixing (Hypotheses H4 and H5). In all conditions, the disease hardly ever spread to more than ten percent of the entire population (six nodes), which was also considerably less than expected by our simulations (see Figure S4 in the Supplementary Information).

Furthermore, Table 4 shows the results of logistic regression analyses for factors that contribute to getting infected. The strongest predictor for getting infected is the number of rounds a participant had more than 12 relations. Having many relations creates many potential transmission routes, but on the other hand, having many relations decreases the chance that an infected neighbor is selected by the NIDT for the opportunity to break that relation. Although Table 4 suggests that risk aversion does not affect the probability to get infected, the overall absence of disease spread does not allow a final judgment whether higher risk aversion affects the probability to get infected in general (Hypothesis H3).

**Table 4:** Risk factors for acquiring infection during one installment of the NIDT.

Constant	−2.004*** (0.333)	
**Risk aversion**		
Risk aversion score	−0.006 (0.008)	
**Parameter settings**		
Clustering (*HI*)	−0.117 (0.092)	
Mixing (*AS*)	0.025 (0.115)	
**Individual-level controls**		
Age	−0.013 (0.008)	
Gender (female)	−0.130 (0.124)	
Gender (other)	0.423 (0.236)	
Mother tongue (English)	−0.073 (0.173)	
Education	−0.018 (0.043)	
COVID-19 concern	−0.035 (0.064)	
COVID-19 positive	0.024 (0.166)	
Rounds relations 12+	0.148*** (0.025)	
Log Likelihood	−1452.84	
AIC	2930	
BIC	3010	
Observations	5758	

The numbers describe a logistic regression model of whether a node acquired an infection during one installment of the NIDT (0: no, 1: yes).

***p < 0.01. SEs in parentheses were made robust using clusters of 2,879 participants and two observations (NIDTs) per participant.

### Neglect of clustering

Table 3 reveals further insights into the decision-making process. We observe significant interaction effects for expected change in clustering and the two clustering conditions. However, contrary to what we would expect, in the high clustering setting decisions were favored that were expected to decrease clustering. In the low clustering setting, decisions were favored that were expected to increase clustering. Considering that in both clustering settings, the baseline networks ensured that all nodes started at an optimal position regarding clustering, a decision neglecting clustering would therefore be likely to work against the initial optimum. Consider, for example, a node with five neighbors and none of them share relations with each other (*LO*). Furthermore, the node gets offered six opportunities of which four refer to neighbors of the node’s neighbors. A random decision that does not consider the expected change in clustering would likely increase clustering.

Furthermore, we performed additional simulations with parameters set according to the experimental data (see Section 2.2 of the Supplementary Information for definitions and Section 3 of the Supplementary Information for a detailed discussion of additional results). These simulations support the expected effect of random decisions on clustering (see Section 3.5 of the Supplementary Information).

### Effects of risk perception

In additional simulations (see Section 2.2 of the Supplementary Information), we increased perceived disease severity and perceived infectivity according to the empirical data. Consequently, artificial agents showed similar avoidance responses towards infected neighbors as participants (see Figure S5 in the Supplementary Information). However, infected nodes are more isolated in the experiment than in the additional simulations. This stronger isolation of infected nodes continues to result in lower infection numbers in the experiment than in the additional simulations (see Figure S6 in the Supplementary Information). Thus, increased risk perception alone cannot explain the discrepancy between the isolation of infected nodes and infection numbers in the experiment and in the simulations.

## Discussion

To study the extent of avoidance behavior in people and how the corresponding level of avoidance affects disease spread in social networks, we developed the *Networking during Infectious Diseases Task* (*NIDT*). Data collected from a large-scale (2,879 participants) online experiment revealed that infected alters are the least desirable relations for susceptible egos and that higher risk aversion causes stronger avoidance of infectious alters (supporting Hypotheses H1 and H2). The comparison of experimental data, initial simulations, and simulations using experimental data suggests that the decisions of participants were driven by higher levels of risk aversion and less focus on increasing point rewards (i.e., not maintaining social network structure). Analyses on decision-making revealed that participants deviated from the agents in our simulations in a few, but important aspects. That is, participants prioritized disease avoidance over social benefits. Especially clustering did not play a role in networking decisions, although it could create higher rewards (up to 20 points per round) than the penalty of being infected (-14 points per round). Thus, clustering deteriorated for both clustering settings (increasing in the low clustering setting, decreasing in the high clustering setting). Additional simulations revealed that increasing risk perceptions alone does not suffice to produce the low infection numbers from the experiment. We found that participants used more sophisticated strategies to avoid infection than the decision-making rules of the simulated agents. That is, participants not only avoided infectious alters, but also susceptible alters with infectious neighbors. Furthermore, infected alters were avoided irrespective of the disease state of egos. That is unexpected because infected and recovered egos were not at risk of experiencing any (additional) disadvantages when neighboring infected alters. Consequently, infected individuals were more isolated in the experiment than in the simulations, causing disease spread to stop quickly in the experiment. This preference for disease avoidance over maintaining social network structure is indispensable to understand and predict human behavior during infectious disease outbreaks. On a theoretical level, these insights ought to be considered for model design. Furthermore, it is an insight we expect to be observable in real-world social networks.

As a result from the strong disease avoidance reactions in our experiment, a fictitious disease could hardly spread to more than ten percent of the nodes in our networks. We could therefore neither confirm nor reject whether higher risk aversion affects the probability to get infected (H3), and whether networks composed of multiple clusters and networks with neighbors similar in risk perception have mitigating effects on disease spread (H4 and H5).

We identify three main reasons for why participants prioritize disease avoidance in networking decisions. First, infections create salient signals (red nodes) with obvious consequences (14 points deducted as penalty for every round of being infected). In comparison to infections, clustering requires more effort to keep track of. That is, determining the number of closed triads requires identifying all neighbors including their relations between each other. Furthermore, changes in clustering may occur stepwise over several rounds, so that corresponding changes in point rewards are less noticeable. In the literature, we typically assume that people act parsimonious regarding cognitive resources^28^, and myopically regarding the optimization of rewards^29^. If a person chooses to optimize only one element, the choice is thus likely in favor of disease avoidance because it is easier to evaluate.

Second, from *Prospect Theory*^30,31^ we know that people do not act rationally in the sense of maximizing rewards purely based on expected economic utility. In particular, people appear to be more motivated by avoiding losses than by maximizing gains. Gains and losses, however, are not absolute outcomes but are relative to a reference point. In addition, people are generally bad at estimating risks or probabilities for the occurrence of events^32^ and tend to overestimate the probability of rare events^33^. As a result, people tend to reject tangible benefits in order to avoid low probabilities of losses^34^. If we consider point rewards from the previous round as a reference point and interpret getting infected as a loss, a deviation of behavior compared to the model is in line with Prospect Theory. That is, participants seem to favor the aversion of a loss (-14 points per round of being infected) above a potential higher benefit (up to 20 points per round for maintaining social structure), although the risk of getting infected is low (15% for one infectious neighbor per round).

Third, the COVID-19 pandemic was ongoing at the time we conducted our experiment in July 2021. Although we did not mention the pandemic in our instructions and kept to a neutral, not emotionalizing language (see Section 1.3 of the Supplementary Information), Slovic & Weber^33^ argue that hazardous events, such as the outbreak of a disease, can trigger *social amplification of risk*^35^. That is, behavioral responses are subject not only to individual risk assessment, but also to *social amplification stations*, such as the media, cultural groups, or interpersonal networks. A topic that passes through the filter of these social amplification stations and is backed up with additional information, such as expert commentary, can trigger responses that go beyond the mitigation of immediate harm. In the context of disease avoidance, the consistent presence of the COVID-19 pandemic in the public mind may therefore have amplified the avoidance of infected nodes in our experiment.

Although our experiment was intentionally conducted in an abstract environment, and its outcomes cannot easily be generalized to the real world, the results can be considered a warning signal that loss of social cohesion might occur during epidemics. Just like in our experiment, visible symptoms of infection, or shared information about personal health are salient signals for behavioral adaptation. Furthermore, the consumption of disease related information in the media may increase perceived disease severity and perceived susceptibility to infectious diseases^36^. Both symptoms of others and media consumption have the potential to trigger or reinforce avoidance reactions in the short term. Corrosion of social cohesion as a result of disease avoidance, however, is a comparatively less salient signal for behavioral adaptation, while it has the potential to lower well-being in the long term. The COVID-19 pandemic has already shown that the implementation of distancing measures can lead to social isolation and consequently, to anxiety, depression, sleep deprivation, substance abuse, etc.^37–41^. An innate preference to avoid infections may reinforce social isolation, lead to the corrosion of social coherence, and thus the loss of individual well-being in the long run.

Apart from the experimental findings, the empirical evaluation of model predictions is itself a noteworthy contribution. The intersection of theory and empirical testing allows the emergence of solid scientific understanding. Here we provide a tangible tool for such integration. Our work therefore fills a critical gap by providing an empirical mechanism for evaluating theoretical models, thus fulfilling a recurring demand in scientific circles for tools that allow a smooth transition from theoretical predictions to empirical validations.

It is furthermore important to point out the limitations of our study. First, although we provide simple rules and give examples of how clustering affects point rewards, keeping track of clustering in the experiment might have been abstract or tedious. However, to some degree, this is also to be expected in real-world settings. As argued before, infections create salient signals with obvious consequences, while deterioration of clustering is a gradual process that is thus more difficult to observe and stop. Second, we acknowledge that risk perception is a combination of analytic and affective processes that heavily rely on each other^32,33^. Our experiment, however, is focused on the analytic processes of risk perception. That is, we used neutral, not emotionalizing language without framing of personal relationships.

As disease spread in the experiment was not sufficient to test our group-level hypotheses, future work could adjust the experiment to overcome this issue. The number of decision opportunities, for example, could be limited, or some relations could be fixed, so that relations with infected neighbors persist longer. This would reflect relations that are maintained independent of the circumstances (e.g., family). Additionally, the visibility of disease states could be modified. That is, disease states could depend on the distance to the ego, with disease states of immediate neighbors the most likely to be revealed. Another option could be the implementation of a delay between acquiring an infection and revealing the infectiousness of a node to reflect that transmission may occur before symptom onset. Furthermore, the experiment might produce random transmission events independent of the network, resembling disease transmissions through short-term, non-personal contacts (e.g., public transport, grocery stores). Finally, future research could explore the other model parameters or different parameter settings to provide a more comprehensive picture of human networking behavior in the context of infectious disease spread. For example, a reduced transmissibility should lead to a lower perceived probability of getting infected, thus weaker avoidance responses, and thus more infected individuals.

In addition, the experiment provides important insights into how the model can be improved conceptually to better capture human behavior. Firstly, adjusting the risk perception parameter (*r*) of susceptible agents using empirical data alone was not sufficient to reproduce the avoidance reactions observed in participants. This can be considered in the model by adding a weight parameter to control the perceived risks of infections (Eq. (3)). Secondly, we observed that about 66% of decisions were made to increase point rewards, while there was a clear preference to avoid infected alters. This can be considered in the model by adding a probability to make decisions in order to increase or decrease point rewards when alters are not infected. Thirdly, we observed that participants avoided infectious alters regardless of their own disease state, resulting in stronger isolation of infected individuals in the experiment than in the simulations. Extending point penalties for a relationship with an infected alter to all agents can thus capture such stronger avoidance responses in the simulations.

In conclusion, we find that despite the similarity in test environments, available actions, and behavioral mechanisms, small but significant differences between human participants and simulated agents have a strong impact on the course of our hypothetical epidemics. However, we do not consider a shortcoming of either method. It rather demonstrates that the combined application of theoretical models and empirical studies provides better insights than either method alone, and should therefore find greater implementation in scientific research.

## Materials and methods

### Experiment

#### Participants

A total of 48 experimental sessions were conducted between July 7 and July 22, 2021. All but one experimental sessions consisted of 60 participants, resulting in a total of 2,879 participants. In the one session that could not be filled up completely, one node remained non-responsive and thus did not perform any actions. All but five participants, who reported technical difficulties (i.e., connection loss, glitches in the user interface), completed the entire study (2,874). Unresponsive nodes remained in the network, but did not initiate any relational changes. We assume that the nodes for this small number of unresponsive participants do not substantially affect the behavior of other participants, nor the main dynamics of the game. Another 2,972 persons signed up for the study, but either did not show up for the corresponding session, or could not be assigned to a session of 60 participants. A session lasted between 24 and 80 minutes (*M* = 47, *SD* = 12). Participants earned between £5.00 and £8.79 (*M* $=\pounds 5.29$, *SD* $=\pounds 0.45$). Demographic data of participants is summarized in Table S8 in the Supplementary Information.

#### Recruitment and compensation

Recruitment was carried out via *Prolific* (https://www.prolific.co/), an online participant recruitment platform for surveys and market research. We used a short registration study (<5 minutes) in which we described the design and background of the experiment, what was expected from participants, possible advantages and disadvantages of participating, the confidentiality of data processing, and provided contact information of the main researcher and independent contacts for comments and complaints about the study (see Section 1.1 of the Supplementary Information).

Furthermore, participants were informed that they earn a minimum of £5.00 per 60 minutes of their participation as required by Prolific. Points earned during the experiment (see Section “Design”) were converted at the exchange rate of 500 points = £1.00, and paid as bonus if the amount exceeded the compensation for the time. Due to complaints about missing or incorrect bonus payments, we extended the instructions with a specific example after the first three sessions. The added example describes that a participant who earned 3,000 points during a 60-minute-long session earned £6.00: £5.00 for the minimum payment and £1.00 in addition as a bonus.

Informed consent was obtained from all participants. That is, by clicking the “Sign up” button, participants declared that they had read all information about the experiment, that they agreed to participate at a specified time, and that they may quit the study at all times without explanation or consequences. Registered participants were invited personally to the experiment at the specified time using their anonymous Prolific ID. The experiment was approved by the Faculty Ethics Review Board (FERB) of the Faculty of Social and Behavioral Sciences of Utrecht University on June 14, 2021 (reference number 21-0210). All experiments were performed in accordance with relevant guidelines and regulations.

#### Design

The experiment followed a two-by-two mixed design. That is, two settings for clustering (low - *LO*; high - *HI*) that were performed within the same experimental session, and two settings for social mixing (random - *RA*; assortative regarding risk aversion - *AS*) that were performed between different experimental sessions. To avoid order bias, we alternated the initial clustering settings every experimental session and the social mixing settings every second experimental session. Consequently, the order of settings per session was as follows: session 1.1: *LO:RA*; session 1.2: *HI:RA*; session 2.1: *HI:RA*; session 2.2: *LO:RA*; session 3.1: *LO:AS*; session 3.2: *HI:AS*; session 4.1: *HI:AS*; session 4.2: *LO:AS*; session 5.1: *LO:RA*; etc.

Settings for clustering differed in the degree of clustering of the baseline network structures, while other properties were kept the same. That is, one network contained a single large cluster (*LO*: global clustering coefficient = 0.06, average degree = 5.93, closeness = 0.975), while the other contained several densely connected clusters (*HI*: global clustering coefficient = 0.62, average degree = 5.93, closeness = 0.955; see Fig. 1).

Settings for social mixing differed in two aspects. First, participants were assigned to nodes depending on individual risk aversion score. To achieve randomly mixed networks (*RA*), participants were assigned to nodes so that neighbors mostly possessed different risk aversion scores. To achieve assortatively mixed networks (*AS*), participants were assigned to nodes so that neighbors mostly possessed similar risk aversion scores. Second, nodes for decision opportunities were selected based on risk aversion score. That is, to inhibit assortative mixing we offered nodes independent of risk aversion score (*RA*: ω = 0.0); while to foster assortative mixing, we prioritized offered nodes that were the most similar regarding risk aversion score (*AS*: ω = 0.8). As Figure S6 in the Supplementary Information shows, this approach was effective in both simulations and experiment.

Furthermore, we predefined the index cases (the initially infected node) for each condition so that the epidemic started with the most average node regarding degree, local clustering coefficient, and risk aversion score.

We then performed a series of simulated experiments (*simulation 1*) with the same setup as intended for the online experiment (48 sessions, 96 games, networks with 60 nodes) to determine parameter settings for homophily (*RA*: ω = 0.0, *AS*: ω = 0.8), infectivity (γ = 0.15), disease severity (σ = 0.34), recovery time ($\tau$ = 4 rounds), number of offered nodes per round (ϕ = 0.2), probability for each offered node to be a neighbor ($\psi$ = 0.5), and probability for each offered node to be a neighbor of a neighbor ($\xi$ = 0.3). Parameters were selected so that differences in clustering and homophily produce epidemics differing in the number of (simultaneously) infected nodes and duration (see *simulation 1* in Figure S4 in the Supplementary Information). Risk perception parameters of the agents were set randomly using a probability distribution, based on data reported by a study using the same task (*M* = 1.27, *SD* = 0.45; rescaled from the reported scores: *M* = 19.7, *SD* = 6.98 to the range used in the simulations: 0.0–2.0)^42^. Based on these settings, agents were more likely to perceive high risks of infection and thus more likely to behave risk-averse.

Finally, we rescaled utility to make point rewards more comprehensible. That is, rather than awarding $b_{1} \cdot t_{i} - (c_{1} \cdot t_{i} + c_{2} \cdot t_{i}^{2}) = 1.0 \cdot 6 - (0.2 \cdot 6 + 0.067 \cdot 6^{2}) = 2.388$ points for the optimal number of 6 relations, we used a factor of 41.55 to award 100 points. The same applies to the reward for proportion of closed triads (e.g., optimal proportion for *LO*: $b_{2} \cdot \left( 1 - 2 \cdot \frac{\left| x_{i} - \alpha \right| }{\max \left( \alpha , 1 - \alpha \right) } \right) = 0.5 \cdot \left( 1 - 2 \cdot \frac{\left| 0.0 - 0.0 \right| }{\max \left( 0.0, 1.0 - 0.0 \right) } \right) = 0.5 * 41.88 = 21$ points) and the penalty for being infected ($\sigma = 0.34 * 41.88 = 14$ points per round being infected).

#### Procedure

An experimental session was composed of three parts: a risk aversion assessment using the *staircase task* (Part I), two installments of the NIDT (Part II), and a survey (Part III; see Fig. 1). All parts were programmed in Elixir^43^, displayed using Phoenix^44^, and performed using a web browser (see Fig. 2). Participants started immediately with Part 1 after clicking a link sent via a personal message through the Prolific platform. Part 2 started when 60 participants had finished Part 1 and finished reading the instructions for Part 2. If after 20 minutes there were not enough participants ready to fill up a network game (≤ 50), all waiting participants were released and paid a show-up fee of £5.00.

The staircase task^21,22^ is a series of binary choices to determine the individual risk aversion score. In five consecutive rounds, participants were asked to choose between either a 50:50 chance to win 300 points vs. 0 points, or a guaranteed reward of a certain number of points. In the first round, the guaranteed reward was 160 points. The guaranteed reward for round two depended on whether a participant opted for the safe choice (80 points) or the gamble (240 points) in round one. According to the staircase task, people who repeatedly opt for the safe choice, although the rewards are dropping, are more risk-averse than others who start to gamble at some threshold. After five rounds, participants end up at a position between 1 and 32 on the staircase, with 1 being the most and 32 the least risk-averse. For an easier comparison with the theoretical model, we inverted and recoded the risk aversion score to a range between 0.0 and 2.0, with higher values denoting higher risk aversion and 1.0 representing risk neutrality.

After completing Part 1, participants were asked to read the instructions for the NIDT (see Section 1.3 of the Supplementary Information). Once 60 participants finished reading the instructions and answered three questions to test their understanding of the rules, participants were assigned randomly to nodes in the network, and played three test rounds to familiarize themselves with the user interface (see Fig. 2) and how to perform the task. Thereafter, the first NIDT started by assigning participants to nodes in the network according to the setting for social mixing and their risk aversion score (see Section “Design”).

Each round of the NIDT consists of four stages: decisions 1 (60 seconds to maintain/dissolve existing relations and propose new relations to other participants), decisions 2 (30 seconds to accept proposals to create new relations), computation of point rewards, and computation of disease transmissions. During the entire time, participants could click a link to open a pop-up window with condensed explanations of the reward system (see Sections 1.4 and 1.5 of the Supplementary Information). Part 2 concluded after two installments of the NIDT. Each NIDT concluded either once no more infected nodes were left, or after a maximum of 20 rounds. For more details on the NIDT, see Section “Introduction” and Fig. 1).

In Part 3, participants were asked to fill in a survey asking for age, gender, mother tongue, level of education, country of residence, COVID-19 concern, and whether tested positive for COVID-19 at some point (see Section 1.6 of the Supplementary Information). By clicking a link to finish the experiment, participants were redirected to Prolific. After all participants finished, the session concluded.

### Data and analysis

Data can be divided into three categories: participant data, network data, and decision data. Participant data contain data for each participant collected only once, consisting of the node IDs in the two networking games, risk aversion score, age, gender, mother tongue, education, residence, COVID-19 concern, and whether being tested positive for COVID-19 at some point. Network data describe the entire network at the beginning of each round in the form of an edge list (session ID, game ID, settings for clustering and social mixing, round number, ID of a node, disease state of that node, ID of a connected node). Decision data contain decisions on whether participants wanted to change relations with offered nodes or not (round number, node IDs receiving offers, node IDs of offered nodes, offer type (relation creation or dissolution), decisions on the relations). From these data, all further data (degree of clustering, homophily, etc.) were computed at the time of analysis.

All data were collected to prevent attribution to individual persons. That is, we received anonymized user IDs from Prolific for compensation purposes only. Furthermore, we stored participant data with our own anonymized user IDs that allowed to match them with network and decision data. All personal information, such as age, gender, education, etc. was provided by the participants actively by filling in the questionnaire at the end of the experiment.

We define the *final size* of an epidemic as the proportion of infected and recovered nodes at the end of a network game. Due to violation of the normality assumptions, we used Wilcoxon rank-sum and Kruskall-Wallis tests to test whether final size differed significantly between the settings for clustering and social mixing. Clustering was computed as the average proportion of closed triads over all possible closed triads per node. In accordance with the concept of degree-based assortative mixing^45^, homophily was defined as the Pearson correlation coefficient of risk aversion score between all pairs of connected nodes. Closeness is defined as the reversed and normalized average distance between any two nodes in the network^46^, p.163].

To understand the decision-making process, we compare the proportions of decisions made to decision opportunities. That is, a node offered (*alter* – either by the system in stage 2 or by another participant in stage 3 of a round) constitutes an opportunity for the receiving node (*ego*) to make a networking decision (create, maintain, dissolve). The decision by the ego to *Create* a relation to an alter is therefore defined as an accepted opportunity to create the corresponding not yet existing relation in both stages 2 and 3. The decision by the ego to *Maintain* a relation to an alter is a declined opportunity to dissolve that relation in stage 2. Finally, the decision by the ego to *Dissolve* a relation to an alter is an accepted opportunity to dissolve that relation in stage 2.

Furthermore, we perform a four-level random intercept logistic regression (level 4: 96 games, level 3: 5,758 nodes, level 2: 52,650 rounds, level 1: 711,159 decisions) on whether a relation is desirable or not. That is, a relation is desirable if the opportunities to create and maintain a relation are accepted. Consequently, a relation is undesirable if the opportunity to create a relation is declined and the opportunity to dissolve a relation is accepted. Additionally, we performed a logistic regression analysis on whether a node got infected during one installment of the NIDT. Thus, results indicate risk factors for acquiring infections. Standard errors were made robust using clusters of 2,879 participants and two observations (NIDTs) per participant. We mean centered risk aversion score and consider linear relationships for predictors in all regression analyses.

### Ethical approval

The experiment was approved by the Faculty Ethics Review Board (FERB) of the Faculty of Social and Behavioral Sciences of Utrecht University on June 14, 2021 (reference number 21-0210). All experiments were performed in accordance with relevant guidelines and regulations. Informed consent was obtained from all participants.

### Supplementary Information

Supplementary Information.

## Data Availability

The simulation data, the Java 8 source code to generate the simulation data (including an executable program and an easy to use graphical user interface), and the R scripts to analyze the data during the current study are available under the GPLv3 license on GitHub (https://www.github.com/hnunner/nidm-simulation). The experiment data are available upon request from the corresponding author.

## References

[1] Davis R, Campbell R, Hildon Z, Hobbs L, Michie S (2015). Theories of behaviour and behaviour change across the social and behavioural sciences: A scoping review. Health Psychol. Rev..

[2] Verelst F, Willem L, Beutels P (2016). Behavioural change models for infectious disease transmission: A systematic review (2010–2015). J. R. Soc. Interface.

[3] Ferguson N (2007). Capturing human behaviour. Nature.

[4] Funk S, Salathé M, Jansen VAA (2010). Modelling the influence of human behaviour on the spread of infectious diseases: A review. J. R. Soc. Interface.

[5] Jones JH, Salathé M (2009). Early assessment of anxiety and behavioral response to novel swine-origin influenza A (H1N1). PLoS ONE.

[6] Bish A, Michie S (2010). Demographic and attitudinal determinants of protective behaviours during a pandemic: A review. Brit. J. Health Psychol..

[7] Leppin A, Aro AR (2009). Risk perceptions related to SARS and avian influenza: Theoretical foundations of current empirical research. Int. J. Behav. Med..

[8] d’Andrea V, Gallotti R, Castaldo N, Domenico MD (2022). Individual risk perception and empirical social structures shape the dynamics of infectious disease outbreaks. PLOS Comput. Biol..

[9] Kitchovitch S, Lì P (2010). Risk perception and disease spread on social networks. Proc. Comput. Sci..

[10] Koku E, Felsher M (2020). The effect of social networks and social constructions on HIV risk perceptions. AIDS Behav..

[11] Badham J, Stocker R (2010). The impact of network clustering and assortativity on epidemic behaviour. Theor. Popul. Biol..

[12] Keeling MJ (1999). The effects of local spatial structure on epidemiological invasions. Proc. R. Soc. B Biol. Sci..

[13] Miller JC (2009). Percolation and epidemics in random clustered networks. Phys. Rev. E.

[14] Nunner H, Buskens V, Kretzschmar M (2021). A model for the co-evolution of dynamic social networks and infectious disease dynamics. Comput. Soc. Netw..

[15] Nunner H, Buskens V, Teslya A, Kretzschmar M (2022). Health behavior homophily can mitigate the spread of infectious diseases in small-world networks. Soc. Sci. Med..

[16] Chang SL, Piraveenan M, Pattison P, Prokopenko M (2020). Game theoretic modelling of infectious disease dynamics and intervention methods: A review. J. Biol. Dyn..

[17] Camerer CF (2003). Behavioural studies of strategic thinking in games. Trends Cogn. Sci..

[18] Lunn PD, Ní Choisdealbha A (2018). The case for laboratory experiments in behavioural public policy. Behav. Public Policy.

[19] Woike JK, Hafenbrädl S, Kanngiesser P, Hertwig R (2022). The transmission game: Testing behavioral interventions in a pandemic-like simulation. Sci. Adv..

[20] Falk A, Heckman JJ (2009). Lab experiments are a major source of knowledge in the social sciences. Science.

[21] Andersen S, Harrison GW, Lau MI, Rutström EE (2006). Elicitation using multiple price list formats. Exp. Econ..

[22] Falk A, Becker A, Dohmen TJ, Huffman D, Sunde U (2016). The preference survey module: A validated instrument for measuring risk, time, and social preferences. IZA Discuss. Papers.

[23] Ormel J, Lindenberg S, Stevererink N, Verbrugge LM (1999). Subjective well-being and social production functions. Soc. Indic. Res..

[24] Jackson MO (2008). Soc. Econ. Netw..

[25] Burt, R. S. *Structural holes: The social structure of competition* (Cambridge, 1992).

[26] Simmel G (1950). The Sociology of Georg Simmel.

[27] Coleman JS (1994). Foundations of Social Theory.

[28] Gigerenzer G, Todd PM (1999). Simple Heuristics That Make Us Smart.

[29] Halevy, N. In *Advances in Experimental Social Psychology* 1–66 (Elsevier, 2016).

[30] Kahneman D, Tversky A (2013). Prospect theory: An analysis of decision under risk. Handb. Fundam. Financ. Decis. Mak. Part.

[31] Tversky A, Kahneman D (1992). Advances in prospect theory: Cumulative representation of uncertainty. J. Risk Uncertain..

[32] Slovic P, Peters E (2006). Risk perception and affect. Curr. Dir. Psychol. Sci..

[33] Slovic, P. & Weber, E. U. Perception of risk posed by extreme events. *Risk Manag. Strat. Uncertain World*, 1–21 (2002).

[34] Denes-Raj V, Epstein S (1994). Conflict between intuitive and rational processing: When people behave against their better judgment. J. Pers. Social Psychol..

[35] Kasperson RE (1988). The social amplification of risk: A conceptual framework. Risk Anal..

[36] Tagini S (2021). It won’t happen to me! Psychosocial factors influencing risk percep tion for respiratory infectious diseases: A scoping review. Appl. Psychol. Health Wellbeing.

[37] Banerjee D, Rai M (2020). Social isolation in COVID-19: The impact of loneliness. Int. J. Social Psychiat..

[38] Heape A (2021). Loneliness and social isolation in older adults: The effects of a pandemic. Perspect. ASHA Spec. Interes. Groups.

[39] Kim HHS, Jung JH (2021). Social isolation and psychological distress during the COVID-19 pandemic: A cross-national analysis. Gerontologist.

[40] Pietrabissa G, Simpson SG (2020). Psychological consequences of social isolation during COVID-19 outbreak. Front. Psychol..

[41] Sepúlveda-Loyola WA (2020). Impact of social isolation due to COVID-19 on health in older people: Mental and physical effects and recommendations. J. Nutr. Health Aging.

[42] Vriens, E. & Buskens, V. *Managing Risk Heterogeneity in Risk-Sharing Groups: A Multi-Method Study on Risk Aversion and Solidarity* (2020) (Accessed 19 May 2022).

[43] Elixir Core Team. *Elixir* version v1.11.3. https://elixir-lang.org/ (2021).

[44] McCord, C. *Phoenix Framework Version* v1.5.3. https://phoenixframework.org/ (2020).

[45] Newman MEJ (2002). Assortative mixing in networks. Phys. Rev. Lett..

[46] Buechel B, Buskens V (2013). The dynamics of closeness and betweenness. J. Math. Sociol..

