# Supplementary material for: Disease avoidance threatens social cohesion in a large-scale social networking experiment
Source: Sci Rep. 2023 Dec 18;13:22586. doi: 10.1038/s41598-023-47556-0 (PMC10730866; doi:10.1038/s41598-023-47556-0)
Supplement: Supplementary file 1 — Supplementary Information. [file 41598_2023_47556_MOESM1_ESM.pdf]

Supplementary Information for

Disease avoidance threatens social cohesion in a large-scale social networking experiment

Hendrik Nunner[1*], Vincent Buskens[2,3], Rense Corten[2,3], Casper Kaandorp[4], Mirjam Kretzschmar[3,5]

[1]: Institute for Multimedia and Interactive Systems (IMIS), University of Lübeck, Germany

[2]: Department of Sociology/ICS, Utrecht University, The Netherlands

[3]: Centre for Complex System Studies (CCSS), Utrecht University, The Netherlands

[4]: Research Data Management Support, Utrecht University, The Netherlands

[5]: Julius Center for Health Sciences and Primary Care,
University Medical Center Utrecht, The Netherlands

[*]: Corresponding author
Institute for Multimedia and Interactive Systems (IMIS), University of Lübeck, Building 64, Ratzeburger Allee 160, 23562 Lübeck, Germany; email: hendrik.nunner@uni-luebeck.de

Contents

1 Communication with participants **1**

1.1 Registration . . . 1

1.2 Registration confirmation . . . 5

1.3 Instructions . . . 6

1.4 Pop-up Game A . . . 11

1.5 Pop-up Game B . . . 13

1.6 Survey . . . 15

1.7 Cannot be assigned . . . 17

2 Additional definitions **18**

2.1 Simulations to determine parameter settings for the experiment . . . 18

2.2 Additional simulations . . . 20

3 Additional results **22**

3.1 Demographic data of the participants . . . 22

3.2 Effect of settings and conditions on epidemics . . . 23

3.3 Avoidance of infected alters 24
3.4 Factors contributing to networking decisions 25
3.5 Determinants of decision-making beyond avoidance behavior 26
3.6 Effect of parameter variations on network structure and epidemic size . . . 28

References **30**

Figures

S1 Illustration of clustering settings 9
S2 Illustration of clustering settings (Pop-up Game A) 12
S3 Illustration of clustering settings (Pop-up Game B) 14
S4 Effects of settings and conditions on epidemics 24
S5 Avoidance of infected alters 25
S6 Progression of properties over time (extended) 27
S7 Parameter variations and their effects in simulations 29

Tables

S1 Chance of infections (instructions). 8
S2 Points per number of relations (instructions). 9
S3 Chance of infections (pop-up Game A). 11
S4 Points per number of relations (pop-up Game A). 11
S5 Chance of infections (pop-up Game B). 13
S6 Points per number of relations (pop-up Game B). 13
S7 Parameter ranges 19
S8 Demographic data of the participants. 22
S9 Countries of residence of the participants. 23
S10 Three-level random-intercept logistic regression of networking decisions. . . 26

1 Communication with participants

1.1 Registration

Dear Sir, Madam,

we would like to invite you to participate in the study "Progression of epidemics in a networking game". This study will help to expand our knowledge of networking behavior in the context of infectious diseases. The study will be conducted on *DATE*, at

- *TIME* BST/IST/WEST (Ireland, Portugal, UK)
- *TIME* CEST (Austria, Belgium, Croatia, Czechia, Denmark, France, Germany, Hungary, Italy, Luxembourg, Malta, Netherlands, Poland, Slovakia, Slovenia, Spain, Sweden)
- *TIME* EEST (Bulgaria, Cyprus, Estonia, Finland, Greece, Latvia, Lithuania, Romania).

You will receive a link to participate in the study about 10 minutes before the specified date and time via Prolific. Since this is an interactive study, you cannot join later than 10 minutes after the specified time.

Design/execution of the study

The form of this experiment is a large-scale interactive browser game imitating the spread of infectious diseases in social networks.

Background of the study

The COVID-19 pandemic has shown that our globally connected world is highly vulnerable to the spread of infectious diseases. This study will help us to understand how social networks change over the course of an epidemic.

What is expected of you as a participant

To participate in the study, you will need a desktop computer or laptop (no smartphones or tablets). With regard to the browser, we advise you to use Google Chrome or Mozilla Firefox. Using Internet Explorer is not recommended, as it may not support all features. Furthermore, we expect you to be available for about 1 hour and 40 minutes. Note that during the experiment, some other members may be waiting until you have completed a

given task, before they can proceed. So, please be mindful of other participants and only sign up if you can complete all tasks in a timely fashion. Please only sign up if you can commit to completing it.

Possible advantages and disadvantages of the study

The study is an interactive browser game. All interactions and diseases are only virtual and do not have any consequences in the real world.

Remuneration/compensation

During the experiment, you can earn points. These points will be converted at the end of the experiment at the exchange rate of:

500 points = £1.00 (€1.17)

The number of points you earn depends on your own choices and the choices of other participants. Depending on your performance in the experiment, you will earn maximally £10.00 (€11.65). We will guarantee you a minimum payment of £5.00 (€5.83) per 60 minutes of your participation as required by Prolific, but if your earned points are beyond the minimum payment, you get the difference as your bonus. *For example, if you earn 3,000 points and the experiment takes 60 minutes, you earn £6.00: £5.00 for the minimum payment and £1.00 in addition as a bonus.*[1] The experiment will take a maximum of 100 minutes (1 hour 40 minutes), probably less. It may happen that you cannot be assigned to a group to participate in the study. In that case, you will receive the minimum amount as announced on Prolific.

Confidentiality of data processing

This study requires us to collect some of your personal data and demographics (age, gender, mother tongue, education, country, marital status, employment, household income). We need these data in order to be able to answer our research questions properly. Any personal data will all be anonymously stored separately from your Prolific ID so that they can never be traced back to you yourself. The computer on which your personal details is stored is secured to the highest standards, and only researchers involved will have access to this data. The data itself will also be protected by a security code. Your data will be stored for at least 10 years. This is in accordance with the guidelines provided by the

[1]Note that this example has been added after the first three sessions, due to many complaints about missing or incorrect bonus payments.

VSNU Association of Universities in the Netherlands. Please refer to the website of the Authority for Personal Data: https://autoriteitpersoonsgegevens.nl/nl/onderwerpen/avg-europese-privacywetgeving, for more information about privacy.

Voluntary participation

Participation in this study is voluntary. You can end your participation in the study at any time, without any explanation and without any negative consequences. If you end your participation, we will use the data collected up to that point, unless you explicitly inform us otherwise.

Questions, independent contact, and complaints officer

If you have any questions about the study, please contact the main researcher, Hendrik Nunner at h.nunner@uu.nl. If you have any comments about the study, please reach out to our independent contact Sanjana Singh at s.singh@uu.nl. If you have an official complaint about the study, you can email the complaints officer at klachtenfunctionaris-fetcsocwet@uu.nl.

Consent

By checking the box and clicking the "Sign up" bottom below, I declare that I have read all information about the study "Progression of epidemics in a networking game" presented on this website and agree to participate in the study on on *DATE*, at

- *TIME* BST/IST/WEST (Ireland, Portugal, UK)
- *TIME* CEST (Austria, Belgium, Croatia, Czechia, Denmark, France, Germany, Hungary, Italy, Luxembourg, Malta, Netherlands, Poland, Slovakia, Slovenia, Spain, Sweden)
- *TIME* EEST (Bulgaria, Cyprus, Estonia, Finland, Greece, Latvia, Lithuania, Romania).

I know that I may at all times quit the study without any explanation or consequences.

With kind regards,

Hendrik Nunner (h.nunner@uu.nl),

Vincent Buskens (v.buskens@uu.nl),

Mirjam Kretzschmar (m.e.e.kretzschmar@umcutrecht.nl),

Rense Corten (r.corten@uu.nl)

Download the information and consent form co-signed by the main researcher here:

DOWNLOAD INFORMATION AND CONSENT FORM

I understand that I can only participate in the study at the specified date with the link that I will receive via Prolific.

SIGN UP

1.2 Registration confirmation

Thank you for signing up for the online academic study “Progression of epidemics in a networking game” on *DATE*, at

- *TIME* BST/IST/WEST (Ireland, Portugal, UK)
- *TIME* CEST (Austria, Belgium, Croatia, Czechia, Denmark, France, Germany, Hungary, Italy, Luxembourg, Malta, Netherlands, Poland, Slovakia, Slovenia, Spain, Sweden)
- *TIME* EEST (Bulgaria, Cyprus, Estonia, Finland, Greece, Latvia, Lithuania, Romania).

Please remember:

- You will receive a link to participate in the study about 10 minutes before the specified date and time via Prolific.
- You cannot join later than 10 minutes after the specified time.
- You will need a desktop computer or laptop (no smartphones or tablets).
- We advise you to use Google Chrome or Mozilla Firefox, as Internet Explorer may not support all features.

BACK TO PROLIFIC

1.3 Instructions

You are now taking part in a decision-making experiment. Please read the following instructions carefully. They contain everything you need to know to participate in the experiment. The data for this study is collected and controlled by Hendrik Nunner of the Utrecht University Institute (EUI). If you have any questions, please email Hendrik Nunner at h.nunner@uu.nl.

During the experiment, you can earn points. These points will converted at the end of the experiment at the exchange rate of:

500 points = £1.00 (€1.17)

The number of points you earn depends on your own choices and the choices of other participants. Depending on your performance in the experiment, you will earn maximally £10.00 (€11.65). We will guarantee you a minimum payment of £5.00 (€5.83) per 60 minutes of your participation as required by Prolific, but if your earned points are beyond the minimum payment, you get the difference as your bonus. *For example, if you earn 3,000 points and the experiment takes 60 minutes, you earn £6.00: £5.00 for the minimum payment and £1.00 in addition as a bonus.*[2] The experiment will take a maximum of 100 minutes (1 hour 40 minutes), probably less. It may happen that you cannot be assigned to a group to participate in the study. In that case, you will receive the minimum amount as announced on Prolific. (Note that this is slightly adapted from the recruitment text, based on our experience and misunderstandings in an earlier session).

At the end of the experiment, you will be shown a message confirming your participation and a link redirecting you to Prolific when clicked on. Clicking this link is required to finalize your participation. Only when you completed all tasks and got redirected to Prolific, you will receive the money you earned during the experiment in your Prolific account.

Please note that you may at all times quit the study without any explanation or consequences.

PLEASE CLICK TO PROCEED

[2]Note that this example has been added after the first three sessions, due to many complaints about missing or incorrect bonus payments.

Part 1

In part 1 of the experiment, you will be confronted with a series of five situations. Each situation requires you to choose between two options. One option is to accept a sure payment of a particular number of points. The second option is to gamble with a 50:50 chance of getting 300 points or getting nothing. Realize that you will get paid according to your choice, and if you decide to gamble, the outcome indeed depends on a random flip of a coin which is executed by the computer.

The number of points you earn for part 1 is the average number of points related to the five decisions you made (score of each situation divided by five).

PLEASE CLICK TO PROCEED

Part 2

Overview

In part 2 of the experiment, you participate in two games. In each game, you are one person in a network of 60 participants. You are displayed as a square on your own screen. All other participants are shown as circles. We call your square and the circles of other participants "nodes". Relations between participants are indicated as lines between the nodes. Each game consists of about 15 to 20 rounds. Before round 1, a starting network is generated on the screen and one participant in the network will be infected with a (hypothetical) disease. The node of this participant will be colored red. Nodes that have relations with an infected (red) node can also get infected in subsequent rounds.

Game rounds

Each game consists of about 15 to 20 rounds. Each round consists of 4 stages.

Stage 1. In this stage, nodes can get infected. In the first round, only one node is infected. In all other rounds, other nodes can get infected. The probability of getting infected depends on the number of red nodes with whom you are connected. If you are not connected with a red node, you cannot get infected. If you are connected to 1 red node, the probability of infection is 0.15 and increases for each additional infected node (see below).

Table S1: Chance of infections (instructions).

# of infectious neighbors	0	1	2	3	4	5	6	7	8	9	10
chance of getting infected	0.00	0.15	0.28	0.39	0.48	0.56	0.62	0.68	0.73	0.77	0.80

If you get infected, your node will also turn red, and you will stay infected for exactly 4 rounds. After these four rounds, your node will turn green, you will get immune, and you cannot get the disease anymore in this game. All other immune nodes will also be colored green.

Stage 2. In stage 2, you can break relations or propose new relations in the network. There will be 12 nodes displayed on the right side of the screen, for which you can either choose to break the relation or to propose a new relation. The buttons to make the decisions are also colored (red if that node is infected with the disease, green if that node is immune and gray otherwise). Breaking relations you can decide yourself, but before you can establish a new relation, the other participant needs to agree on this relationship as well. You will have exactly 60 seconds to make your decisions in this stage in each round. After that, the game proceeds automatically.

Stage 3. In stage 3, you can indicate which relations that others have proposed to you, you indeed also would like to have. These are again displayed on the right side of the screen and colored as explained before. Other participants will simultaneously decide whether to accept your proposals.

Stage 4. In stage 4, your points for this round are calculated (see Earning points).

Note that stages 1 (disease transmission) and 4 (points computation) are done by the system and the outcomes will be presented as information on the screen (color of nodes, number of points). So in practice, you will move back and forth between stages 2 (breaking / proposing relations) and 3 (accepting proposed relations).

Earning points

How many points you earn depends on three things:

1. **Your relations:** If you have 0 relations, you earn 0 points. The first relation earns you quite some points, the second a bit less, and so on until you can earn maximally 100 points for 6 relations. If you have more than 6 relations, the number of points you earn will decrease again.

Table S2: Points per number of relations (instructions).

# of relations	0	1	2	3	4	5	6	7	8	9	10	11	12	13	...
points per round	0	31	56	75	89	97	100	97	89	75	56	31	0	0	...

2. **Relations between nodes connected to you:** The two games you play will differ in how many points you earn based on the relations between your connections. The game you are playing will be indicated on the screen.

Game A You will be awarded 20 additional points if there are no relations between any of the nodes you are connected to. For each relation between these nodes, you will lose some of these 20 points.

Game B You will be awarded 20 additional points if you are connected to 5 other nodes that all have relations among each other as well, while you have one relation to another node not in this group. If your connections have fewer relations among each other, you lose some of these 20 points.

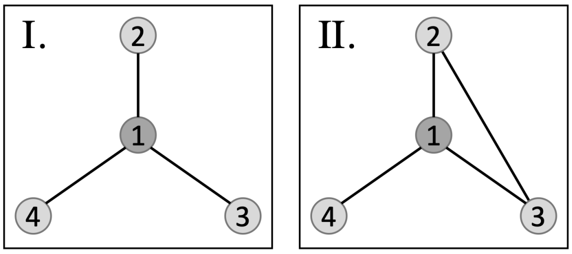

Figure S1: **Illustration of clustering settings.** (I.) No relation between the connections of node 1. (II.) One relation between connections (2 and 3) of node 1, while one connection (4) is to a node not in this group.

3. **Having the disease:** 14 points will be deducted from the points you earn from your relations for each round that you are infected.

User interface

Figure 2 from the main text was shown accompanied by an instructional video to explain the elements of the user interface. The video is available upon request.

Final notes

We will first run a trial game with 3 rounds, in which you can try out the interface. You do not earn anything in the trial game, but you can get a feel for the game and experience how you earn points. After that, we start with the first paid game.

If you have questions or do not understand something, please email the main researcher Hendrik Nunner at h.nunner@uu.nl. Otherwise, click the continue button on the screen.

PLEASE CLICK TO PROCEED

1.4 Pop-up Game A

Disease transmission

The probability of getting infected depends on the number of infected (red) nodes with whom you are connected:

Table S3: Chance of infections (pop-up Game A).

# of infectious neighbors	0	1	2	3	4	5	6	7	8	9	10
chance of getting infected	0.00	0.15	0.28	0.39	0.48	0.56	0.62	0.68	0.73	0.77	0.80

Infected nodes turn red (including your own node) and remain infected for 4 consecutive rounds. After 4 rounds, infected nodes turn green (including your own node), become immune, and can therefore not get infected again.

Earning points in Game A

How many points you earn depends on three things:

1. **Your relations:** The number of points you can earn depends on the number of relations you have. Six relations is optimal. Fewer or more relations will result in fewer points.

Table S4: Points per number of relations (pop-up Game A).

# of relations	0	1	2	3	4	5	6	7	8	9	10	11	12	13	...
points per round	0	31	56	75	89	97	100	97	89	75	56	31	0	0	...

2. **Relations between nodes connected to you:** You will be awarded 20 additional points if there are no relations between any of the nodes you are connected to. For each relation between these nodes, you will lose some of these 20 points.

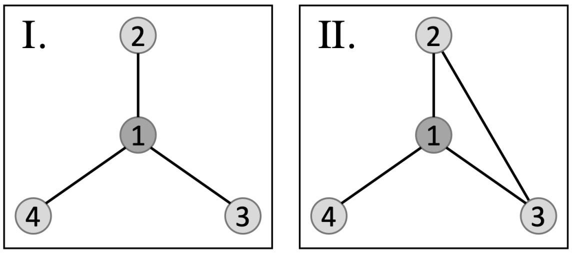

Figure S2: **Illustration of clustering settings (Pop-up Game A).** (I.) No relation between the connections of node 1. (II.) One relation between connections (2 and 3) of node 1, while one connection (4) is to a node not in this group.

3. **Having the disease:** 14 points will be deducted from the points you earn from your relations for each round that you are infected.

1.5 Pop-up Game B

Disease transmission

The probability of getting infected depends on the number of infected (red) nodes with whom you are connected:

Table S5: Chance of infections (pop-up Game B).

# of infectious neighbors	0	1	2	3	4	5	6	7	8	9	10
chance of getting infected	0.00	0.15	0.28	0.39	0.48	0.56	0.62	0.68	0.73	0.77	0.80

Infected nodes turn red (including your own node) and remain infected for 4 consecutive rounds. After 4 rounds, infected nodes turn green (including your own node), become immune, and can therefore not get infected again.

Earning points in Game B

How many points you earn depends on three things:

1. **Your relations:** The number of points you can earn depends on the number of relations you have. Six relations is optimal. Fewer or more relations will result in fewer points.

Table S6: Points per number of relations (pop-up Game B).

# of relations	0	1	2	3	4	5	6	7	8	9	10	11	12	13	...
points per round	0	31	56	75	89	97	100	97	89	75	56	31	0	0	...

2. **Relations between nodes connected to you:** You will be awarded 20 additional points if you are connected to 5 other nodes that all have relations among each other as well, while you have one relation to another node not in this group. If your connections have fewer relations among each other, you lose some of these 20 points.

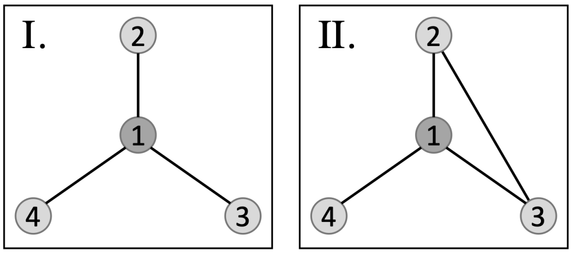

Figure S3: **Illustration of clustering settings (Pop-up Game B).** (I.) No relation between the connections of node 1. (II.) One relation between connections (2 and 3) of node 1, while one connection (4) is to a node not in this group.

3. **Having the disease:** 14 points will be deducted from the points you earn from your relations for each round that you are infected.

1.6 Survey

- What is your age? *NUMBER FIELD*
- What is your gender? *DROP-DOWN FIELD*

  1 Female

  2 Male

  3 Other

- What is your mother tongue? *DROP-DOWN FIELD*

  1 English

  2 Other

- What is your highest completed level of education? *DROP-DOWN FIELD*

  1 No formal education

  2 Primary school (elementary education)

  3 Lower secondary (secondary completed does not allow entry to university: obligatory school)

  4 Upper secondary (programs that allows entry to university)

  5 Post secondary, non-tertiary (other upper secondary programs toward labor market or technical formation)

  6 Lower level tertiary, first stage (also technical schools at a tertiary level)

  7 Upper level tertiary (Master, Doctor)

  8 No answer

- What is your country of residence? *DROP-DOWN FIELD – PHONE COUNTRY CODES*

  93 Afghanistan

  355 Albania

  213 Algeria

  ...

  263 Zimbabwe

- How concerned were/are your to attract COVID-19?

1 Very

2 A bit

3 Hardly

4 Not at all

- Did you get COVID-19?

0 No

1 Yes

1.7 Cannot be assigned

Dear participant,

thank you for participating in the academic study "Progression of epidemics in a networking game"! As explained in the instructions, participants are assigned to groups. This means that when not enough people show up to form a group of 60, not everybody gets to participate.
Unfortunately, you could not be assigned to a group.
The minimum payment as announced in the study will be transferred to you shortly. Please make sure to exit the study by clicking the "Back to Prolific" button below.

Kind regards,
Hendrik Nunner

BACK TO PROLIFIC

2 Additional definitions

2.1 Simulations to determine parameter settings for the experiment (*simulation 1*)

Determining parameter settings for the experiment was a four-step process. In the first step, we selected conditions (*LO:RA*, *LO:AS*, *HI:RA*, *HI:AS*) and fixed parameters for each setting (clustering: *LO*, *HI*; social mixing: *RA*, *AS*) that we deemed feasible for the money available to conduct the experiment. Our goal was to use the largest networks possible with enough sessions to achieve sufficient statistical power. Our time limit was 90 minutes per session (resulting in at least £7.50 per participant), with two installments of the NIDT per session. Within this time limit, we estimated about 20 minutes for reading the instructions, about 30 minutes per NIDT, and a 10-minute buffer. For a single installment of the NIDT, we estimated 1 minute for stage 1 and 30 seconds for stage 2 of each round. Consequently, each installment of the NIDT had to stop after a maximum of 20 rounds. Preliminary simulations showed that parameter settings presented in Table S7 and settings for risk perception parameters that were taken from a previous study by Vriens & Buskens [1], which used the same staircase task to measure risk avoidance[3], provided the best results. That is, the disease had sufficient time to spread, so that after 20 rounds most simulations had no more infected nodes in the network. Furthermore, simulations created epidemics of different size depending on settings for clustering and social mixing.

In the second step, we selected the baseline networks used for the two clustering settings. That is, we first generated 50 networks with a preferred proportion of 0.0 closed triads, and 50 networks with a preferred proportion of 0.667 closed triads. The remaining parameters were set as presented in Table S7. To make sure that the initial networks were as similar as possible, except for clustering, we then selected one predefined network for each clustering setting (see the flowchart of the experiment in Fig. 1 of the main text) so that they had the same largest available average degree (5.933) and the closest available value for closeness (*LO*: 0.975, *HI*: 0.955). Furthermore, we defined fixed orders for assigning participants to nodes based on their risk aversion score. That is, neighbors in the random social mixing setting have mostly different risk perception settings, while neighbors in the assortative mixing setting have mostly similar risk perception settings.

[3]We rescaled the reported scores of $M = 19.7$ and $SD = 6.98$ to $M = 1.27$, $SD = 0.45$ to match the risk perception parameter in the simulations.

Table S7: Parameter ranges used for network generation and determination of game parameters, as well as settings used for game parameters (*LO*, *HI*, *RA*, *AS*).

Parameter	Range	Setting	Use
Utility/points			
I. Social network			
Benefit per social tie*	$b_1 = 1.0$	1.0	all
Simple cost per tie*	$c_1 = 0.2$	0.2	all
Marginal cost per tie*	$c_2 \in \{0.1, 0.067, 0.05\}$	0.067	all
Benefit for triadic closure	$b_2 = 0.5$	0.5	all
Preferred proportion of closed triads†	$\alpha \in \{0.0, 0.667, 0.75\}$	0.0<br>0.667	*LO*, *RA*, *AS*<br>*HI*, *RA*, *AS*
II. Disease			
Disease severity	$\sigma > 0$	0.34	all
Infectivity	$0 \leq \gamma \leq 1$	0.15	all
Recovery time in time steps	$\tau \in \mathbb{Z}^+$	4	all
Homophily			
Likelihood of ties similar in risk perception	$0 \leq \omega \leq 1$	0.0<br>0.8	*LO*, *HI*, *RA*<br>*LO*, *HI*, *AS*
**Network / offerings**			
Network size	$N \in \{12, 24, 40, 60, 80\}$	60	all
Proportion of N offered per time step	$0 \leq \phi \leq 1$	0.2	all
Proportion of ϕ as distance 1 ties	$0 \leq \psi \leq 1$	0.5	all
Proportion of ϕ as distance 2 ties	$0 \leq \xi \leq 1$	0.3	all

Notes: *: The combination of $b_1 = 1.0$, $c_1 = 0.2$, and $c_2 \in \{0.1, 0.067, 0.05\}$ sets the preferred number of ties to 4, 6, and 8 respectively. †: The setting of $\alpha = 0.667$ with a preferred number of 6 ties ($c_2 = 0.067$) realizes a preference of 4 out of 6 neighbors sharing connections.

In the third step, we simulated the entire data collection, with 48 experimental sessions and two installments of the NIDT per experimental session. We also made sure that the agents between the two installments of the NIDT were not reinitialized with new risk perception parameters, but only assigned to the network positions according to the social mixing setting. The results of these simulations are denoted in the results as *simulation 1*.

In the fourth step, we adjusted point rewards to create easy-to-understand rules. For this, we took the optimum of 6 ties as the basis and rather than awarding $b_1 \cdot t_i - (c_1 \cdot t_i + c_2 \cdot t_i^2) = 1.0 \cdot 6 - (0.2 \cdot 6 + 0.067 \cdot 6^2) = 2.388$ points, we used a factor of 41.55 to award 100 points. Rewards for clustering and costs for being infected were rescaled accordingly.

2.2 Additional simulations (*simulation 2*)

To get a better insight on how decision-making differed between the agents in our initially simulated experiments (*simulation 1*) and participants in the experiment, we performed an additional series of simulated experiments (*simulation 2*). We used the same setup as in the experiment, with as many parameters set according to data from our experiment as possible. That is, we set risk perception (parameter r in equation 3 of the main text) to the risk aversion score observed in our experiment (M = 1.22, SD = 0.46), which was slightly less risk-averse than the initial setting (M = 1.27, SD = 0.45) based on Vriens & Buskens [1]. Furthermore, we found that 65.61% of all decisions in the experiment were rewarding in terms of increasing or maintaining point rewards, while there was a clear preference to avoid infected alters. We therefore adjusted agents so that all decisions that involved infected alters were made in order to increase point rewards, while two out of three decisions that did not involve infected alters were made to increase point reward and one out of three decisions was made to decrease point reward. We also found that 14% of the participants tried to maximize the number of relations rather than point rewards. We, therefore, initialized 14% of the agents with lower marginal costs for relations so that they sought to attain twenty ($c_2 = 0.02$) rather than six ($c_2 = 0.067$) relations. Finally, to achieve a similarly strong avoidance reaction towards infected alters, we increased perceived disease severity and perceived infectivity by a factor of 2.5 (see Fig. S5 **(a)** and Fig. S7).

Two settings, however, could not be immediately set so that we had to extend our model in two ways. First, we added a parameter that allows to set the probability of making a networking decision that decreases (rather than increases or maintains) point rewards ($\epsilon = 0.66$):

- Repeat until all decision opportunities have been processed:
  - If alter is infected or $\epsilon <= U[0, 1]$:
    - Make decision to maximize point rewards using equation 1 of the main text
  - else:
    - Make decision to decrease point rewards using equation 1 of the main text

Second, we extended the third term of equation 1 of the main text not only with a risk perception parameter ($0.0 < r_i < 2.0$) for susceptible agents as done in simulation 1 (see equation 3 of the main text), but also with an overestimation factor (η):

$$\left[\sigma\right] \rightarrow (\pi_i^{2-r} \cdot \sigma^r) \cdot \eta \tag{S1}$$

We set η to a value of 2.5 to achieve a similar strong avoidance reaction of susceptible agents towards infected agents, as observed in the experiment (see Section 3.3). Note that we used an overestimation factor rather than inflating risk parameter (r). That is because inflating r would, on the one hand, require a value (1.8) that impedes variation between agents ($0.0 \leq r \leq 2.0$) without creating a long not observed tail towards lower values. On the other hand, artificially inflated risk scores do not correspond to the scores measured using the staircase task.

3 Additional results

3.1 Demographic data of the participants

Table S8: Demographic data of the participants.

	%	N
Gender		
Female	41.2	1,185
Male	54.5	1,570
Other	1.0	30
No answer	3.3	94
Age		
18 to 29	72.0	2,072
30 to 39	16.7	482
40 to 49	6.1	177
50 to 59	2.7	79
60 and above	0.6	18
No answer	1.8	51
Mother tongue		
English	16.5	475
Other	80.8	2,325
No answer	2.7	79
Education		
No formal education	0.1	2
Primary school	1.0	28
Obligatory school	4.0	114
Secondary (allowing entry to university)	42.3	1,219
Tertiary (Bachelor, Maser, PhD)	49.5	1,424
No answer	3.2	92
COVID-19 concern		
Very	27.9	802
A bit	47.4	1,365
Hardly	13.8	398
Not at all	8.7	250
No answer	2.2	64
COVID-19 positive (at some point)		
Yes	13.8	397
No	84.4	2,429
No answer	1.8	53

Table S9: Countries of residence of the participants.

	%	N
Portugal	22.5	647
Poland	21.2	609
UK	14.2	408
Italy	12.1	348
Spain	6.4	183
Greece	5.9	171
Hungary	2.6	76
Ireland	1.8	53
France	1.6	45
Estonia	1.4	41
Netherlands	1.3	38
Czech Republic	1.2	35
Slovenia	1.1	31
Germany	1.0	29
Belgium	0.9	25
Latvia	0.8	24
Sweden	0.8	23
Finland	0.7	21
Austria	0.2	5
Denmark	0.1	4
Luxembourg	0.1	3
USA/Canada/Caribbean	0.1	1
Thailand	0.1	1
New Zealand	0.1	1
No answer	2.0	57

3.2 Effect of settings and conditions on epidemics

Figure S4 shows an extended version of Fig. 4 in the main text. As discussed in the main text, we observed no significant differences in outcome measures (final size, duration, peak size) in the experiment. In simulation 1, however, the simulation performed to determine parameter settings for the experiment, we observe smaller and lower peaking epidemics for the baseline network consisting of multiple clusters (column 1). Furthermore, increase in social mixing shows a tendency for smaller, shorter, and lower peaking epidemics (column). In combination (column 3), low clustering and random mixing produce on average the largest, longest, and highest peaking epidemics, while high clustering and assortative mixing produce on average the smallest, shortest, and lowest peaking epidemics. After adjusting the parameter settings to data from the experiment (simulation 2), we observe that the epidemics remain on average larger, longer, and higher peaking as compared to the experiment, while the effect of conditions on epidemics disappears.

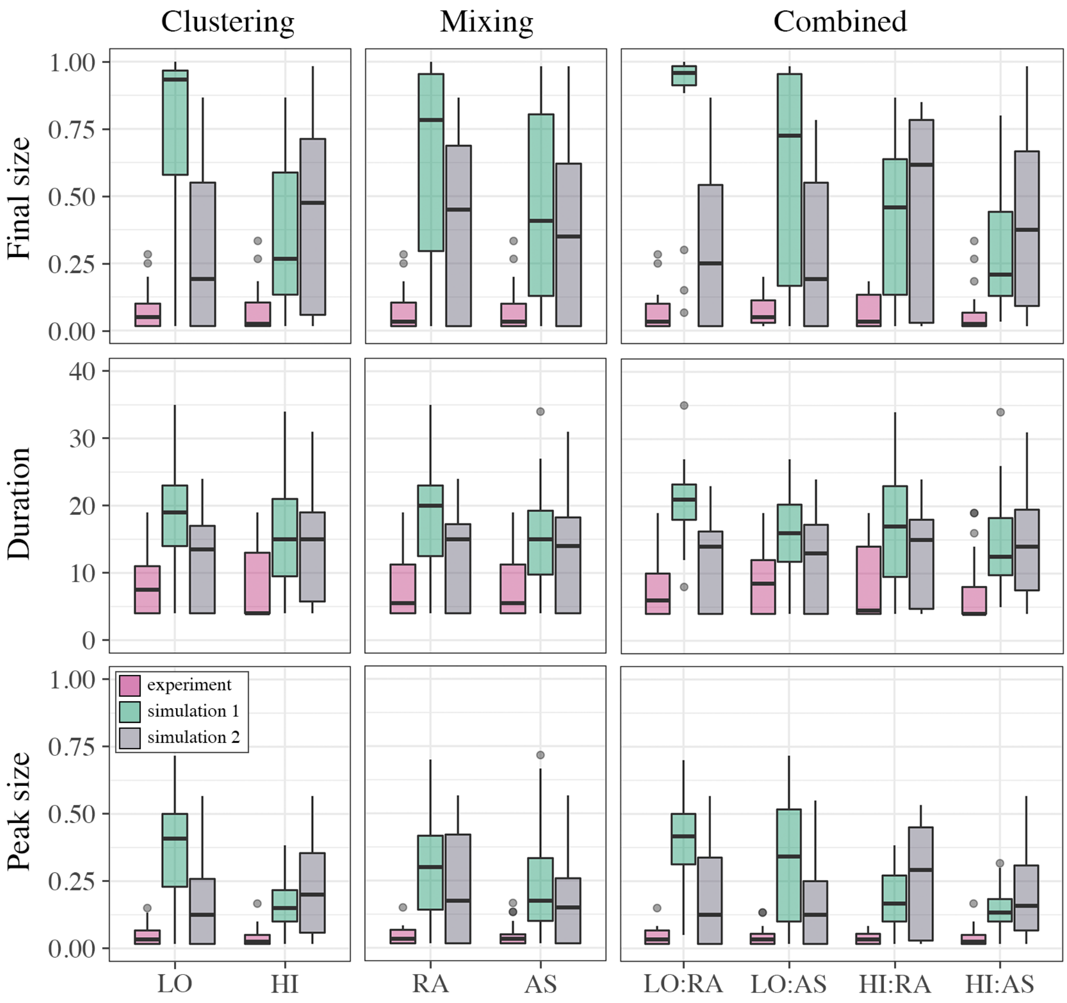

Figure S4: **Effects of settings and conditions on epidemics.** Box-and-whisker plots show the median, interquartile range, minimum, maximum, outliers of final size (proportion of cumulatively infected nodes; row 1), duration (in rounds; row 2), and peak size (maximum proportion of nodes infected at the same time; row 3) by settings for clustering (column 1) and social mixing (column 2), as well as conditions (column 3).

3.3 Avoidance of infected alters

Figure S5 shows how quickly susceptible egos disconnected from infected alters depending on the number of infected neighbors of the ego (**(a)**) and how this affects the average degree of infected nodes over the course of an infection (**(b)**). We see that avoidance of infected nodes is much stronger in the experiment than in simulation 1. By increasing the perceived risk of getting infected and perceived severity of the disease by a factor of 2.5, we increase the avoidance behavior of agents to a degree similar to participants in the experiment (see Fig. S5 **(a)**). This alone, however, does not suffice to achieve a similar strong isolation of infected nodes as observed in the experiment (see Fig. S5 **(b)**).

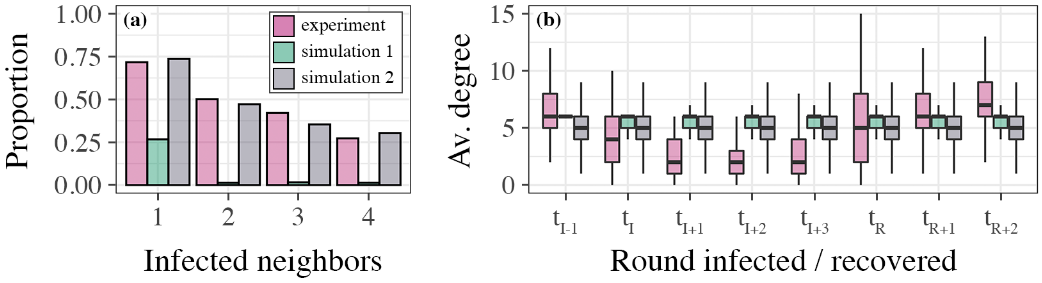

Figure S5: **Avoidance of infected alters.** Plot **(a)** shows the proportion of performed relation dissolutions to dissolution opportunities of susceptible egos and infected alters depending on the number of infected neighbors. Plot **(b)** shows the average degree of nodes from one round before infection (t_{I-1}) until two rounds after recovery (t_{R+2}). Both plots are divided by data source (pink: experiment, green: simulation 1, purple: simulation 2).

3.4 Factors contributing to networking decisions

Table S10 shows a comparison of factors contributing to networking decisions. Model 1 describes factors contributing to the attractiveness of a relation. We define a relation to be attractive as a binary variable composed of decisions to create a relation (proposals in stage 1 and accepted proposals in stage 2 of a round) and declined opportunities to dissolve a relation. Models 2, 3, and 4 describe factors contributing to whether a decision opportunity (proposing a relation to an alter in stage 1 of a round, accepting a proposal of an alter in stage 2 of a round, dissolving a relation) was taken.

Table S10: Three-level random-intercept logistic regression of networking decisions.

	Model 1 (rel. attractive)	Model 2 (rel. proposed)	Model 3 (proposal accepted)	Model 4 (rel. dissolved)
Constant	5.866^{***} (0.074)	1.050^{***} (0.115)	3.013^{***} (0.160)	-5.927^{***} (0.133)
**Disease states**				
Ego^S - Alter^S without infected neighbor	-0.103^{*} (0.059)	-0.522^{***} (0.088)	-0.217 (0.135)	0.025 (0.107)
Ego^S - Alter^S with infected neighbor	-1.070^{***} (0.061)	-1.350^{***} (0.091)	-0.593^{***} (0.138)	1.532^{***} (0.108)
Ego^S - Alter^I	-3.728^{***} (0.067)	-3.626^{***} (0.108)	-3.732^{***} (0.149)	4.523^{***} (0.118)
Ego^S - Alter^R	1.070^{***} (0.063)	1.018^{***} (0.092)	0.600^{***} (0.145)	-1.060^{***} (0.128)
Ego^I - Alter^S without infected neighbor‡	0.200^{***} (0.062)	-0.035 (0.090)	0.199 (0.233)	
Ego^I - Alter^S with infected neighbor	-0.014 (0.079)	0.157 (0.113)	1.398^{***} (0.488)	0.447^{***} (0.130)
Ego^I - Alter^I	-1.347^{***} (0.094)	-0.307^{**} (0.144)	-1.119^{***} (0.222)	2.367^{***} (0.139)
Ego^I - Alter^R	1.949^{***} (0.114)	2.282^{***} (0.148)	1.479^{***} (0.347)	-0.500^{*} (0.270)
Ego^R - Alter^S without infected neighbor	-0.886^{***} (0.057)	-1.059^{***} (0.087)	-0.744^{***} (0.132)	0.779^{***} (0.107)
Ego^R - Alter^S with infected neighbor	-0.813^{***} (0.083)	-0.884^{***} (0.122)	-0.578^{***} (0.176)	1.078^{***} (0.163)
Ego^R - Alter^I	-1.560^{***} (0.084)	-1.411^{***} (0.150)	-1.136^{***} (0.171)	1.730^{***} (0.139)
Ego^R - Alter^R (reference)				
**Risk aversion**				
Risk aversion score (RAS)	0.185 (0.172)	0.602^{***} (0.226)	0.406 (0.403)	0.648^{*} (0.340)
Ego^S - Alter^S without infected neighbor × RAS	-0.037 (0.161)	-0.445^{**} (0.210)	-0.189 (0.402)	-0.636^{*} (0.337)
Ego^S - Alter^S with infected neighbor × RAS	-0.130 (0.179)	-0.611^{**} (0.240)	-0.220 (0.437)	-0.604^{*} (0.355)
Ego^S - Alter^I × RAS	-0.833^{***} (0.235)	-1.429^{***} (0.392)	-1.664^{***} (0.510)	-0.083 (0.425)
Ego^S - Alter^R × RAS	0.182 (0.199)	-0.366 (0.252)	0.157 (0.505)	-0.889^{*} (0.533)
**Network properties**				
Degree	-0.492^{***} (0.007)	-0.435^{***} (0.010)	-0.465^{***} (0.018)	0.613^{***} (0.017)
Degree^2	0.020^{***} (0.0004)	0.020^{***} (0.001)	0.022^{***} (0.001)	-0.022^{***} (0.001)
Degree of alter	-0.043^{***} (0.001)	-0.030^{***} (0.002)	-0.040^{***} (0.003)	0.027^{***} (0.003)
Clustering (*HI*)	0.026 (0.032)	-0.035 (0.043)	-0.213^{***} (0.056)	0.007 (0.037)
HI × ECC†	-10.258^{***} (0.106)	-10.570^{***} (0.142)	-14.200^{***} (0.277)	8.032^{***} (0.252)
LO × ECC†	6.655^{***} (0.137)	3.872^{***} (0.151)	2.491^{***} (0.287)	-27.789^{***} (0.461)
**Decision opportunity types**				
Number of offers by opportunity type	-0.098^{***} (0.003)	-0.029^{***} (0.005)	-0.342^{***} (0.009)	-0.045^{***} (0.007)
Creation opportunity in stage 1 of a round	-4.126^{***} (0.010)			
Creation opportunity in stage 2 of a round	-3.299^{***} (0.015)			
Log Likelihood	−286,474	−153,387	−43,380	−74,957
AIC	573,006	306,827	86,815	149,967
BIC	573,339	307,119	87,065	150,240
Observations	711,159	360,493	79,360	271,306
Number of groups (rounds)	52,650	52,650	39,403	52,441
Number of groups (nodes)	5,758	5,758	5,736	5,758
Number of groups (games)	96	96	96	96
Variance (rounds)	0.28	0.47	0.31	0.31
Variance (nodes)	0.87	1.65	1.25	0.98
Variance (games)	0.01	0.01	0.04	0.01

Notes: $^{***}p < 0.01$, $^{**}p < 0.05$, $^{*}p < 0.1$, SEs in parentheses. †*ECC*: Expected Change in Clustering. Models are three-level random-intercept logistic regression of whether (Model 1) a relation is attractive (0: declined opportunities to create a relation in stage 1 and 2 of a round, and accepted opportunities to break a relation, 1: accepted opportunities to create a relation in stage 1 and 2 of a round, and declined opportunities to break a relation), (Model 2) an opportunity to create a relation in stage 1 was accepted, (Model 3) an opportunity to create a relation in stage 2 was accepted, and (Model 4) an opportunity to dissolve a relation was accepted.

3.5 Determinants of decision-making beyond avoidance behavior

To delve deeper into the decision-making process, we ran additional simulations with parameters set according to the empirical data (for details see Section 2.2).

Figure S6 shows a comparison of how average utility, average degree, average clustering, average degree of homophily, and disease states progress over time between the experiment (pink), the initial simulation to determine parameter settings for the exper-

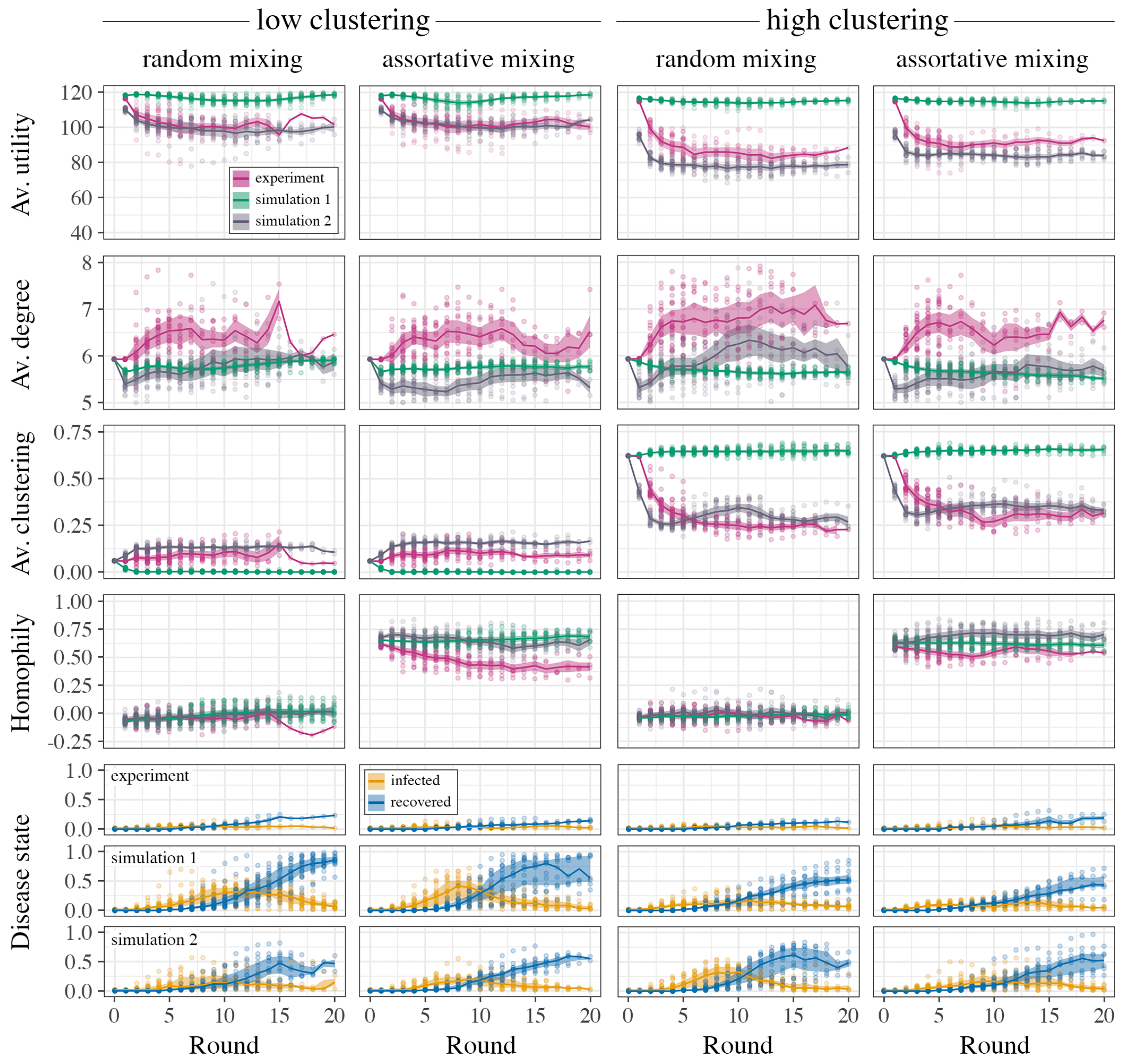

Figure S6: **Progression of properties over time (extended).** Plots are divided by different properties (rows) and settings for clustering and social mixing (columns). Average utility (row 1), average degree (row 2), average clustering (row 3), and degree of homophily (row 4) depict the results for the experiment (pink), simulation 1, which has been performed before the experiment (green), and experiment 2 with parameter settings taken from the experiment (purple). Progression of disease states has been divided into individual rows per data source (experiment: row 4, simulation 1: row 5, simulation 2: row 6). Each plot shows the progression of the corresponding property over time (max. 20 rounds). Dots within the plots show the mean of all nodes per single NIDT. Lines depict the mean and ribbons the standard deviation for NIDT means.

iment (*simulation 1*, green), and the additional simulated experiment with parameters set according to the data from the experiment (*simulation 2*, purple). Furthermore, we divided the plots into the two settings for clustering (columns 1 and 2: LO, columns 3 and 4: HI) and the two settings for social mixing (columns 1 and 3: RA, columns 2 and 4: AS). The plots show that participants did not act like the agents in simulation 1. That is, the average degree was on average higher for both settings, while clustering was on

average higher in the low clustering setting and lower in the high clustering setting. As a consequence, average utility was on average lower. Although 14% of agents that sought to maximize the number of relations, the average degree did not deviate much from the optimal average of six relations.[4] Similar to simulation 1, the average degree of infected nodes remained at a comparatively high level in simulation 2, and thus infected nodes were not as strongly avoided as in the experiment (see Fig. S5, plot (b)). Furthermore, we can see that simulation 2 was very close to the experiment in terms of average clustering. That is, due to two out of three decisions being made randomly if no infected neighbor is involved, we see a rise of clustering in the low clustering setting and a drop of clustering in the high clustering setting. Although the parameter adjustments in simulation 2 produced better matches for average clustering and average utility, they did not affect the spread of the disease.

3.6 Effect of parameter variations on network structure and epidemic size

Figure S7 shows the relationships between parameters that have been set in simulation 2 based on the data collected in the experiment and affected outcome measures (i.e., average degree, average clustering, final size of epidemics). The data is based on 8,000 additional simulations with random variations of marginal costs for the number of relations (c_2), the probability to make a random rather than utility maximizing decision (ϵ), and the factor overestimating the perceived risk of getting infected and the perceived severity of an infection (η). We observe that higher marginal costs, and lower probabilities of random decisions have negative effects on the number of relations. Furthermore, completely random decisions ($\epsilon \approx 1.0$) result in an average clustering of around 0.2 in both clustering settings, while decisions based fully only utility maximization ($\epsilon \approx 0.0$) result in higher average clustering in the *HI* setting ($M = 0.61$, $SD = 0.02$) than in the LO setting ($M = 0.16$, $SD = 0.04$). Finally, higher values for risk overestimates reduce the final size. Additionally, diseases do not spread at all when agents perceive the risk of getting infected and the severity of the disease about three times the unaltered perceived value ($\eta = 3.0$).

[4] Refer to Fig. S7, for an overview of how variations of the input parameters informed by the experiment data in simulation 2 (i.e., marginal costs for additional relations, probability of random decisions, and disease overestimate) affect outcomes (i.e., average degree, average clustering, final size).

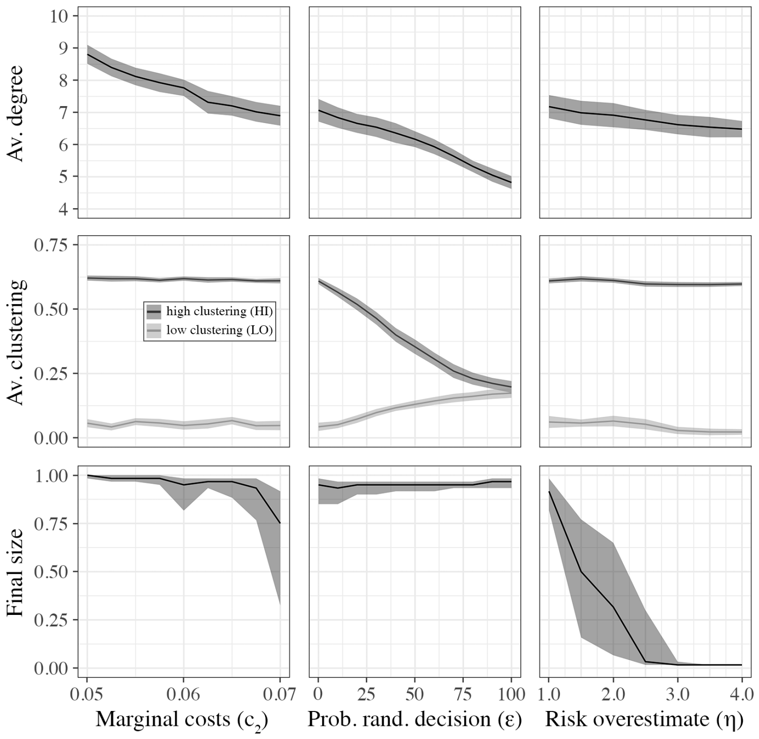

Figure S7: **Parameter variations and their effects in simulations.** Plots are divided by different outcome measures (rows) and simulation parameters (columns). Average clustering is divided into the two clustering settings (LO, HI). Lines depict means, ribbons standard deviations of the corresponding outcome measure.

References

1. Vriens, E. & Buskens, V. *Managing risk heterogeneity in risk-sharing groups: A multi-method study on risk aversion and solidarity* Accessed: 2022-05-19. 2020.
